# Indigenous Microorganisms Offset Arbuscular Mycorrhizal Fungi-Induced Plant Growth and Nutrient Acquisition Through Negatively Modulating the Genes of Phosphorus Transport and Nitrogen Assimilation

**DOI:** 10.3389/fpls.2022.880181

**Published:** 2022-05-09

**Authors:** Wenda Ren, Yun Guo, Xu Han, Yan Sun, Qing Li, Bangli Wu, Tingting Xia, Kaiping Shen, Pan Wu, Yuejun He

**Affiliations:** ^1^Forestry College, Research Center of Forest Ecology, Guizhou University, Guiyang, China; ^2^Key Laboratory of Karst Georesources and Environment, Ministry of Education, Guizhou University, Guiyang, China

**Keywords:** arbuscular mycorrhizal fungi, indigenous microorganisms, nutrients metabolism, phosphorus transport, nitrogen assimilation

## Abstract

Arbuscular mycorrhizal (AM) fungi that promote plant growth and nutrient acquisition are essential for nutrient-deficient karst areas, while they inevitably regulate host plants jointly with indigenous microorganisms in natural soil. However, how indigenous microorganisms regulate AM-induced benefits on plant growth and nutrient acquisition remains unclear. In this study, the *Bidens tripartita* as the common plant species in the karst region was cultivated into three soil substrates treated by AM fungi inoculation (*AMF*), AM fungi inoculation combining with indigenous microorganisms (*AMI*), and the control without AM fungi and indigenous microorganisms (*CK*). The plant biomass and concentration of nitrogen (N) and phosphorus (P) were measured, and the transcriptomic analysis was carried out using root tissues. The results showed that AM fungi significantly enhanced the plant biomass, N, and P accumulation with the reduction of plants’ N/P ratio; however, the indigenous microorganisms offset the AM-induced benefits in biomass and N and P acquisition. In addition, there are 819 genes in differentially expressed genes (DEGs) of *AMF* vs. *AMI* ∩ *AMF* vs. *CK*, meaning that AM fungi induced these genes that were simultaneously regulated by indigenous microorganisms. Furthermore, the enrichment analysis suggested that these genes were significantly associated with the metabolic processes of organophosphate, P, sulfur, N, and arginine biosynthesis. Notably, 34 and 17 genes of DEGs were related to P and N metabolism, respectively. Moreover, the indigenous microorganisms significantly downregulated these DEGs, especially those encoding the PHO1 P transporters and the glnA, glutamate dehydrogenase 2 (GDH2), and urease as key enzymes in N assimilation; however, the indigenous microorganisms significantly upregulated genes encoding PHO84 inducing cellular response to phosphate (Pi) starvation. These regulations indicated that indigenous microorganisms restrained the N and P metabolism induced by AM fungi. In conclusion, we suggested that indigenous microorganisms offset nutrient benefits of AM fungi for host plants through regulating these genes related to P transport and N assimilation.

## Introduction

As functional microorganisms, arbuscular mycorrhizal (AM) fungi can form symbiont with roots of 80% of the terrestrial plants in the world (van der Heijden et al., 2015). It is well established that AM fungi can induce genome-wide reprogramming of host plants, thereby triggering a series of events (Guether et al., 2009), such as nutrient metabolism (Ruzicka et al., 2011; Nanjareddy et al., 2017). Especially, AM fungi can induce the genes related to nutrient transport and assimilation to assist host plants in absorbing and utilizing more nitrogen (N) and phosphorus (P) (Smith and Smith, 2011; Bonneau et al., 2013; Ruiz-Lozano et al., 2016). N is the mineral nutrient element with the greatest demand for plant growth and development (Martinez-Feria et al., 2018), and AM fungi were reported to induce the expression of ammonium transporters MtAMTs in *Medicago truncatula* (Breuillin-Sessoms et al., 2015) and to induce the expression of ${\text{NO}}_{3}^{-}$ transporter NRT in tomato (Ruzicka et al., 2011), thereby facilitating the N acquisition of the host plant. In addition, AM fungi can also regulate the assimilation of N in host roots through increasing the activity of glutamine synthase (GS) (Subramanian and Charest, 1998; Perez-Tienda et al., 2014). Compared to N, the benefits of AM fungi on P availability are more marked (Karandashov and Bucher, 2005). Chen et al. (2017) demonstrated an active and complex P metabolism in mycorrhizal roots of *Poncirus trifoliate* (L.), which involved P transport and acid phosphatase activity regulation. AM fungi can induce the expression of mycorrhiza-specific phosphate (Pi) transporters, such as StPT3 in potato and ZEAma and Pht1 and Pht6 in maize (Javot et al., 2007), respectively. In addition, AM fungi can regulate root architecture (Xia et al., 2020) and produce extensive mycelium for exploring rhizosphere scale (Shen et al., 2020) to help plants absorb nutrients, which are the essential mechanisms for plants to adapt to some special habitat, such as karst habitat with nutrient deficiency owning to thin and barren soil layer (Li et al., 2002). Conversely, the host plant provides sugars and lipids to the fungal partner, such as AM fungi (Jiang et al., 2017; Rich et al., 2017). Thus, the prosperity root activity induced by AM fungi will bring more active and complex soil microorganisms around the rhizosphere soil.

The AM fungi can powerfully influence the properties of rhizosphere, thereby recruiting an overwhelming number of indigenous microorganisms (Philippot et al., 2013). On the one hand, the root extensive mycelium of AM symbiont is an active area of indigenous microorganisms (Gahan and Schmalenberger, 2015), demonstrating an intimate interrelation between AM fungi and other microbes, such as indigenous microorganisms. On the other hand, AM mycelium can transfer the photosynthetic carbohydrate from plant to soil, and this process can attract abundant microorganisms (Drigo et al., 2010; Kaiser et al., 2015). Conversely, the ecological function of AM fungi and the symbiotic process between AM and host plant are also positively affected by indigenous microorganisms through cooperation (Frey-Klett et al., 2007; Ortiz et al., 2015) or negatively affected through competition (Harrier and Watson, 2004; Leigh et al., 2011). On the one hand, previous studies have shown that AM fungi and some indigenous microorganisms, such as rhizosphere bacteria, have a positive cumulative effect on promoting plant growth and resistance to stress (Camprubí et al., 1995; Kasim et al., 2012; Agnolucci et al., 2015). This effect shows that AM fungi and indigenous microorganisms have a potential cooperative relationship in promoting plant growth. On the other hand, due to the obligate requirements of microorganisms for space and host-derived compounds, the competition between AM fungi and indigenous microorganisms for inner root space, infection sites, and photosynthetic products of the host plant is not surprising (Azcón-Aguilar and Barea, 1997; Harrier and Watson, 2004; Vigo et al., 2010). Therefore, the competition can probably inhibit AM fungi colonizing to plant roots. In addition, other indigenous microorganisms may regulate host plants jointly with AM fungi. For example, Kistner et al. (2005) discovered seven *Lotus japonicas* genes involved in nodulations and in the colonization process of AM fungi. Ohkama-Ohtsu and Wasaki (2010) indicated that the symbiosis between plants and AM fungi might involve the same signaling pathway of the symbiosis between plants and rhizobia. Pathogens can regulate mineral nutrient transporter, which is also regulated by AM fungi, thereby influencing pathogen proliferation, biochemical defense of host, and related signal transduction mechanisms (Sun et al., 2021). Therefore, indigenous microorganisms in the soil are recognized as the third party involved in AM fungi regulation for the host plant, not just soil-borne “free riders” (Jansa et al., 2013); the cooperation and competition between AM fungi and indigenous microorganisms are inevitable in natural soil.

In summary, AM fungi can induce reprogramming on nutrient metabolism to assist host plants in absorbing and utilizing nutrients; meanwhile, indigenous microorganisms are recognized as the third party involved in AM fungi regulation for the host plant (Bonfante and Anca, 2009). Therefore, indigenous microorganisms may positively or negatively affect the AM benefit for the host plant through cooperation or competition (Harrier and Watson, 2004; Frey-Klett et al., 2007; Leigh et al., 2011; Ortiz et al., 2015) in the complex environmental conditions caused by the high heterogeneity of the karst areas. Karst is a typical terrestrial ecosystem characterized by nutrient deficiency and fragile habitat inducing low plant productivity (Liu et al., 2009); the higher heterogeneity of karst habitat provides more complex niches for species-rich plants and indigenous microorganisms as well as AM fungi (Gorbushina, 2007; Lian et al., 2010). In the primary succession of degraded karst ecosystem, some pioneer species, such as Compositae species *Bidens tripartita*, probably establish symbiosis colonized by mycorrhizal fungi accompanying with indigenous microorganisms, which is of great significance to vegetation restoration and soil improvement (Anding et al., 2017). However, how indigenous microorganisms regulate AM-induced benefits on growth and nutrients for these pioneer plants in karst soil remains unclear at present. Therefore, to explore the molecular mechanism of plant adaptability regarding indigenous microorganisms regulating mycorrhizal fungi function for host plant nutrients, an experiment was conducted by a pioneer herb plant of *B. tripartita* from karst habitat cultivated into different microbial soil, including AM fungi accompanying with indigenous microorganisms, and the transcriptomic analysis was carried out in this study. Lin et al. (2017) and Wang et al. (2017) found in their previous studies the positive effect of AM fungi on the accumulation of mineral nutrients and the growth of host plants, while Leigh et al. (2011) demonstrated a competitive relationship between AM fungi and indigenous microorganisms. Volpe et al. (2016) also found possible offsets of indigenous microorganisms on AM fungi benefits triggered by this competition. We hypothesized that AM fungi can promote the growth and the nutrients’ acquisition for *B. tripartita*, while the indigenous microorganisms can offset these benefits (H1); indigenous microorganisms negatively modulate AM-induced nutrient metabolism genes to offset the benefits of AM fungi on plant growth and nutrient accumulation (H2). Furthermore, applying the significance of this research provides the theoretical basis for mycorrhizal technology in vegetation restoration of degraded karst ecosystem and other similar ecorestoration, as well as data support for the plant utilization in functional genes screening through *de novo* transcriptome of *B. tripartita*.

## Materials and Methods

### Experimental Treatment

A potting experiment was conducted by planting *B. tripartita* in polypropylene plastic pots in a plastic greenhouse of Guizhou University campus. The *B. tripartita* seeds were collected from a typical karst habitat in Huaxi, Guiyang City of Guizhou Province, China (E:106°22′ E; N:29°49′ N; 1,120 m above the sea level). *B. tripartita* seeds were planted into three substrates treated by AM fungi inoculation (*AMF*), AM fungi inoculation combining with indigenous microorganisms (*AMI*), and the control without AM fungi and indigenous microorganisms (*CK*). At first, karst limestone soils (Calcaric Regosols, FAO) (He et al., 2019) as cultivation substrates were collected from the surface of plant seeds’ collection sites. The collected karst soil sieved to remove residual roots and stones would be mixed and subsequently divided into two parts, approximately two-third of which were sterilized at 126°C, 0.14 MPa for 2 h to eliminate microbes as sterilized soil for the *CK* and *AMF* treatments. The remaining one-third of the soil was not sterilized as original soil to keep the indigenous microorganism for the *AMI* treatment. Subsequently, 2.3 kg of the sterilized soil or the original soil was taken into a plastic pot (180 mm in diameter × 160 in height). Five seeds of *B. tripartita* were disinfected with 10% H_2_O_2_ solution for 10 min, washed repeatedly in sterile water, and finally sown into each pot. After sowing seeds in each pot, seeds were covered with 200 g of the respective soil according to the treatment in order to keep the germination environment around the seeds moist to promote seed germination. Particularly, the *AMF* treatment used sterilized soil that was inoculated with 10 g AM inoculum of the fungus *Glomus mosseae*, which contained approximately more than 100 spores per gram of soil after propagation; the *AMI* treatment induced through the original soil by adding the extra 10 g inoculum of *G. mosseae* for AM fungi combining with indigenous microorganisms in this experiment. Especially, 10 g of sterilized inoculum was taken into sterilized soil as *CK* treatment soil, and 10 ml of the filtrate by the AM inoculum was taken into the substrate to maintain the microflora consistency except for the target AM fungus *G. mosseae* in this experiment. The experimental design contained three treatments, and each treatment was replicated eight times as 24 pots were placed randomly and regularly during the experiment. After seedling germination, each pot was retained one seedling with normal growth. Plant materials were harvested after 90 days of cultivation, and then the relevant parameters were measured.

In addition, before the soil treatment of this experiment, AM fungi spores were isolated from the collected soil and subsequently morphologically identified by the method of Blaszkowski et al. (2012) with an Olympus BX51 light microscope. Microscopy presented that *G. mosseae* had the largest spore richness as the dominant species in the original soil. Therefore, the *G. mosseae* was selected for this experiment. The *G. mosseae* inoculum was initially purchased from the Institute of Nutrition Resources, Beijing Academy of Agricultural and Forestry Sciences (No. BGA0046) and then propagated for 4 months with *Trifolium incarnatum*.

Furthermore, at harvest, the fresh rhizosphere soil with well-mixed in each pot was stored in a liquid-N tank to determine the composition of fungi and bacteria in cultivated soil. Subsequently, the DNA of soil microorganisms was extracted by E.Z.N.A.^®^ soil DNA kit (Omega Bio-Tek, Norcross, GA, United States). The 16S rRNA gene was PRC-amplified with the primer set 338F (5′-ACTCCTACGGGAGGCAGCAG-3′) and 806R (5′-GGACTACHVGGGTWTCTAAT-3′) targeting the V3-V4 hypervariable regions (Jiang et al., 2019). The ITS region of fungi was PRC-amplified with the primer set ITS1F and ITS2R (Hoch et al., 2019). The amplified products were extracted and purified paired-end sequencing by Illumina MiSeq PE300 platform/NovaSeq PE250 platform (Illumina, San Diego, United States). The raw 16S rRNA and ITS gene sequencing reads were demultiplexed, quality-filtered, and merged to obtain the effective tags. The effective tags were operational taxonomic units (OTUs) clustered with 97% similarity. The taxonomy of each OTU representative sequence was analyzed by RDP Classifier version 2.2 against the 16S rRNA database (Silva 132) and ITS (Unite 7.2) using a confidence threshold of 0.7. The community composition of fungi and bacteria at the genus level is presented in Supplementary Figure 1.

### Determination of the Biomass, the Root Mycorrhizal Colonization, and the Concentration of N and P

The *B. tripartita* seeds were first harvested, and the roots were washed with distilled water. Notably, 300 mg fresh roots collected from 3 replicates with consistent growth in each treatment were frozen in liquid N and transferred to a −80°C refrigerator for subsequent total RNA extraction. The root mycorrhizal colonization rate was determined by intersecting the gridline method of Giovannetti and Mosse (1980); the harvested *B. tripartita* seeds were dried by roots, stems, and leaves, at 105°C for 48 h, and then were weighted for biomass; nutrient concentrations were determined by the Kjeldahl method for N concentration and the molybdenum-antimony anti-colorimetric method for P concentration, referred from Bao (2000). The acquisitions of N and P were calculated through the nutrient concentration multiplying by plant biomass *via* respective roots, stems, and leaves.

### RNA Extraction, cDNA Library Construction, and Illumina Sequencing

Fresh roots of 3 replicates from three treatments were previously stored in a −80°C refrigerator and then a total of 9 fresh roots were used to extract total RNA by using Plant RNA Purification Reagent for plant tissue according to the manufacturer’s instructions (Invitrogen), and genomic DNA was removed using DNase I (TaKara). The quality of the extracted RNA was evaluated using the 2100 Bioanalyzer (Agilent) and was quantified by the ND-2000 (NanoDrop Technologies). Of note, 5 μg high-quality RNA sample was used to construct the sequencing library by an Illumina TruSeq™ RNA Sample Preparation Kit (San Diego, CA, United States). mRNA was enriched according to the polyA selection method by oligo (dT) beads and then fragmented by fragmentation buffer. The fragments of 200 bp were taken as templates, and double-stranded cDNA was synthesized using a SuperScript double-stranded cDNA synthesis kit (Invitrogen, CA) with random hexamer primers and then subjected to end repair, phosphorylation, and addition of “A” base to an adapter. After purification and fragment sorting, the sorted products were amplified by PCR to obtain the library. The libraries were quantified using QuantiFluor^®^ dsDNA System, and nine libraries were sequenced on an Illumina HiSeq X Ten (Illumina, San Diego, CA, United States), and the raw data were obtained.

### *De novo* Assembly and Function Annotation

After removing reads containing adapters, reads with ambiguous ‘‘N’’ bases, and low-quality reads in the raw reads, clean reads were obtained, and their GC content and Q20 were computed. Due to the unavailability of a reference genome of *B. tripartita*, trinity^1^ was used to *de novo* assemble the clean reads for the full transcriptome (Grabherr et al., 2011). Due to the alternative splicing, the assembled transcripts were redundant to obtain unigenes. The assembly integrity of the transcriptome was assessed by Benchmarking Universal Single-Copy Orthologs (BUSCO)^2^ (Simao et al., 2015), and then high-quality data of each sample were mapped back onto the assembly transcripts. Gene ontology (GO) annotation of unigenes was using BLAST2GO version 2.5.0 with a significance threshold of E < 10^–5^. Annotated unigenes were using DIAMOND version 0.8.37.99 with an E < 10^–5^ for the NCBI, Nr, Swiss-Prot, and COG databases to get comprehensive gene information. KOBAS version 2.1.1 was used to compare unigenes with The Kyoto Encyclopedia of Genes and Genomes (KEGG) database to obtain the KO number and its specific biological pathways.

### Quantitation of Gene Expression Levels and Bioinformatics Analysis

Transcripts per million (TPM) reads values, the levels of genes expression, were calculated using RSEM software (Li and Dewey, 2011). The differential expression of the three treatments was analyzed by R package “DESeq2” in using the table of TPM values, and the threshold of significantly differential expression was set as *q* < 0.01 and |*log*_2_(*foldchange*)| > 1 (Love et al., 2014). The *q*-value was obtained by adjusting the *P*-value with the Benjamini and Hochberg (BH) method. GO enrichment analysis of differentially expressed genes (DEGs) was adopted by Goatools^3^ (Klopfenstein et al., 2018); GO terms with corrected *P* < 0.05 were significantly enriched. The KEGG pathways enrichment analysis was analyzed on the free online platform of Majorbio Cloud Platform^4^ with KOBAS^5^ (Xie et al., 2011) whose calculation principle is the same as the GO enrichment analysis, and the threshold setting was the same as the GO enrichment analysis.

### Statistical Analysis

All data were tested for normality and homogeneity of variance before analysis and were analyzed by the one-way ANOVAs adopted by the least significant difference (LSD) among the three treatments of *AMI*, *AMF*, and *CK* at *P* ≤ 0.05, to compare the differences in root mycorrhizal colonization, biomass, N and P accumulations, and N/P ratio. All statistical analyses were performed using the SPSS version 25.0 software, and all graphs were drawn using R version 4.0.4.

## Results

### The Plant Biomass Production, the Root/Shoot Ratio, and the Root Mycorrhizal Colonization of *Bidens tripartita* Under Different Treatments

In Table 1, the significant *AMF* > *AMI* > *CK* of biomass of root, shoot, and individual plant biomass is presented, indicating that the plant biomass was increased by AM fungus when comparing *AMF* with *CK* and *AMI* with *CK*. Especially, a significant *AMF* > *AMI* indicated that indigenous microorganisms decreased the positive effect of AM fungus according to the comparison of *AMF* and *AMI*. The root/shoot ratio of *B. tripartita* under the *CK* treatment was significantly higher than the *AMF* and *AMI* treatments, while *AMF* and *AMI* treatments were not significantly different. The difference of root mycorrhizal colonization between *AMF* and *AMI* treatments was not significant; however, the root mycorrhizal colonization of the *CK* treatment was zero; meanwhile, the AM fungi spore and mycelium were not found in *CK* soil substrate *via* the microscopic detection. In total, AM fungi inoculation significantly promoted the biomass accumulation of *B. tripartita*, while the indigenous microorganisms suppressed the AM-induced benefits on biomass accumulation.

**TABLE 1:** Plant biomass, root/shoot ratio, and mycorrhizal colonization of *B. tripartita* under different treatments.

Treatment	Shoot biomass (g⋅pot^–1^)	Root biomass (g⋅pot^–1^)	Individual plant biomass (g⋅pot^–1^	R/S	Mycorrhizal colonization (%)
*AMF*	3.60 ± 0.79a	1.32 ± 0.29a	4.9323 ± 1.05a	0.37 ± 0.04b	64.60 ± 2.60a
*AMI*	0.65 ± 0.15b	0.29 ± 0.09b	0.9459 ± 0.24b	0.44 ± 0.06b	67.40 ± 2.88a
*CK*	0.04 ± 0.01c	0.03 ± 0.01c	0.0684 ± 0.02c	0.65 ± 0.14a	0 ± 0c

*AMF, AM fungi treatment; AMI, indigenous microorganism combining with AM fungi treatment; CK, sterilized soil control. The letters within column following the mean ± SD (n = 5) indicate significant differences at p < 0.05. The same as below.*

### The Nutrient Concentration of N and P and N/P Ratio of *Bidens tripartita* Under Different Treatments

**FIGURE 1:**
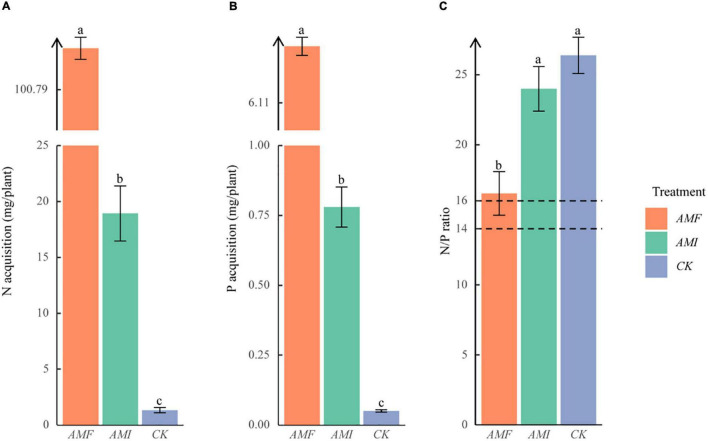
Nutrient acquisition of *B. tripartita* on nitrogen (N) **(A)** and phosphorus (P) **(B)**; N/P ratio of *B. tripartita* seedlings **(C)**. *AMF* = arbuscular mycorrhizal (AM) fungi inoculation treatment; *AMI* = AM fungi inoculation combining with indigenous microorganisms treatment; *CK* = the control without AM fungi and indigenous microorganisms. The different lowercase letters (a, b, and c) above the column indicate significant differences between *AMF*, *AMI*, and *CK* (*P* < 0.05). The dashed lines indicate the N/P ratio equal to 14 and equal to 16, respectively.

The significant *AMF* > *AMI* > *CK* on the nutrient concentration of N and P were presented (Figures 1A,B). In addition, the N/P ratios of the *AMF*, *AMI*, and *CK* treatments were 16.5, 23.9, and 26.4, respectively. However, the N/P ratio of the *AMF* treatment was significantly lower than the *AMI* and *CK* treatments. At the same time, there was no significant difference between *AMI* and *CK* treatments (Figure 1C), which indicated that AM fungi could significantly increase the nutrient absorption of host plants to alleviate the P limitation caused by the karst soil. At the same time, these benefits were decreased due to the indigenous microorganisms.

### Quality of the RNA-Seq and *de novo* Assembly

To reveal the transcriptome responses of *B. tripartita* roots under three treatments, the RNA-seq analysis was performed. Three replicates of *AMF*, *AMI*, and *CK* treatments in a total of 9 cDNA libraries of root tissues were used for paired-end sequencing. There were 52, 54, and 53 million clean reads filtered out in *AMF*, *AMI*, and *CK* treatments, respectively. The range of GC content was from 40.93 to 45.57%, and the lowest clean Q30 bases rate was 95.46% (Table 2). These results indicated that the quality-controlled clean data met the quality requirements for subsequent analysis.

**TABLE 2:** Results of RNA sequencing and mapping.

Sample ID	Raw reads	Clean reads	Clean reads rate (%)	Raw Q30 bases rate (%)	Clean Q30 bases rate (%)	GC content (%)	Mapping ratio (%)
*AMF 1*	50,755,990	50,223,716	98.95	95.28	95.98	44.37	75.04
*AMF 2*	52,067,054	51,686,062	99.26	95.40	95.86	43.92	76.09
*AMF 3*	53,810,542	53,367,560	99.17	95.28	95.79	43.92	76.33
*AMI 3*	57,411,758	56,918,132	99.14	95.11	95.7	40.92	75.41
*AMI* 2	50,750,122	50,365,378	99.24	95.42	95.88	43.36	73.87
*AMI* 3	55,559,726	55,192,138	99.33	95.04	95.46	42.80	74.29
*CK* 1	56,582,836	55,690,938	98.42	94.39	95.93	44.83	73.57
*CK* 2	51,734,854	50,852,052	98.29	94.12	95.79	45.57	76.68
*CK* 3	53,086,744	52,225,052	98.37	94.06	95.75	45.49	76.66

*AMF, AM fungi inoculation treatment; AMI, AM fungi inoculation combining with indigenous microorganisms; CK, the control without AM fungi and indigenous microorganisms.*

Using the clean reads from three treatments, *de novo* transcriptome assembly with Trinity generated 127,699 unigenes and 199,348 transcripts with an average length of 824 and 942 bp. The overview of the assembly is shown in Table 3. The length of unigenes ranged from 201 to 14,089 bp, accompanied by an N50 of 1,304 bp (Supplementary Table 1). Among these treatments, 75.82, 74.52, and 75.64% of reads in *AMF*, *AMI*, and *CK* libraries could be uniquely mapped into the unigenes (Table 3).

**TABLE 3:** Details of the *de novo* transcriptome assembly.

Feature	Unigenes	Transcripts
Total sequence number	127,699	199,348
Total sequence base	105,290,958	187,852,042
GC percent (%)	39.72	39.64
Largest length (bp)	14,089	14,089
Smallest length (bp)	201	201
Average length (bp)	824.52	942.33
N50 length (bp)	1,304	1,417
E90N50 length (bp)	2,058	1,854

### Transcriptome Annotation

Compared with the corresponding database through the above function annotation software, the functions of these unigene sequences can be inferred. A total of 74,394 (59.41%) unigenes were annotated by at least one of the GO, KEGG, COG, NR, Swiss-Prot, and Pfam databases (Supplementary Table 2). In addition, 51.91% of annotated unigenes were allocated to *Helianthus annuus*, which belongs to the Compositae as well as *B. tripartita* (Supplementary Figure 1), indicating that the transcriptome annotation was available for subsequent analysis.

The GO annotation results indicated that 52,318 (41.78%) unigenes were identified and assigned to 53 GO terms under biological process (BP), cellular component (CC), and molecular function (MF); the “cellular process,” “cell part,” and “binding” were the most dominant GO terms (Supplementary Figure 2A). Simultaneously, 35,876 unigenes were annotated to the KEGG database, and 27 pathways were assorted based on six branches. The top three numbers of unigenes were allocated to the second category of “Translation,” “Carbohydrate metabolism,” and “Folding, sorting and degradation” (Supplementary Figure 2B).

### Identification of Differentially Expressed Genes in Roots

**FIGURE 2:**
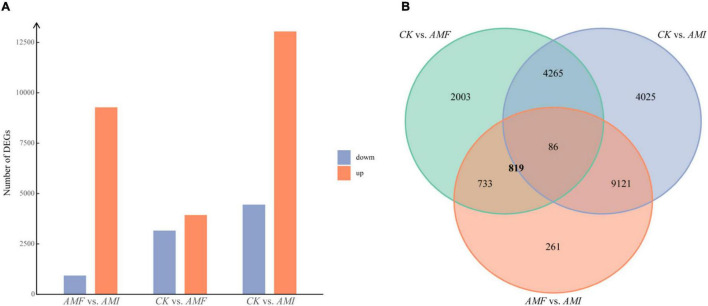
**(A)** The number of DEGs identified in the pairwise comparisons among the three treatments. The thresholds are *q* < 0.01 and |*log*_2_⁡*FoldChange*| > 1. The red and blue columns indicate the numbers of upregulated and downregulated genes in the pairwise comparisons, respectively. **(B)** Venn diagram of the pairwise comparisons among the three treatments. *AMF* vs. *AMI* indicates that compared with the *AMI*, the genes upregulated or downregulated in the *AMF*; *CK* vs. *AMI* indicates that compared with the *CK*, the genes upregulated or downregulated in the *AMI*; *CK* vs. *AMF* indicates that compared with the *CK*, the genes upregulated or downregulated in the *AMF*.

The DEGs among the three treatments were identified by DESeq2 with *q* < 0.01 and |*log*_2_⁡*FoldChange*| > 1. Compared to the *CK* treatment, the *AMF* treatment induced 7,087 DEGs, which contained 3,929 upregulated genes and 3,158 down-regulated genes; compared to the *CK* treatment, the *AMI* treatment induced 17,497 DEGs, which contained 13,045 upregulated genes and 4,452 downregulated genes; compared to the *AMF* treatment, the *AMI* treatment induced 10,201 DEGs, which contained 9,278 upregulated genes and 923 downregulated genes (Figure 2A). To reveal the mechanism of how indigenous microorganisms regulated plant roots to inhibit the AM-induced benefits, the Venn analysis of the DEGs among the three treatments was used to identify the part of DEGs induced by AM fungi inoculation and regulated by indigenous microorganisms. A total of 819 genes overlapped with DEGs of *AMF* vs. *AMI* ∩ *AMF* vs. *CK* (Figure 2B), indicating that these genes were induced by AM fungi and were also regulated by indigenous microorganisms.

### Gene Ontology Enrichment Analysis of Arbuscular Mycorrhizal-Induced Genes Regulated by the Indigenous Microorganism

**FIGURE 3:**
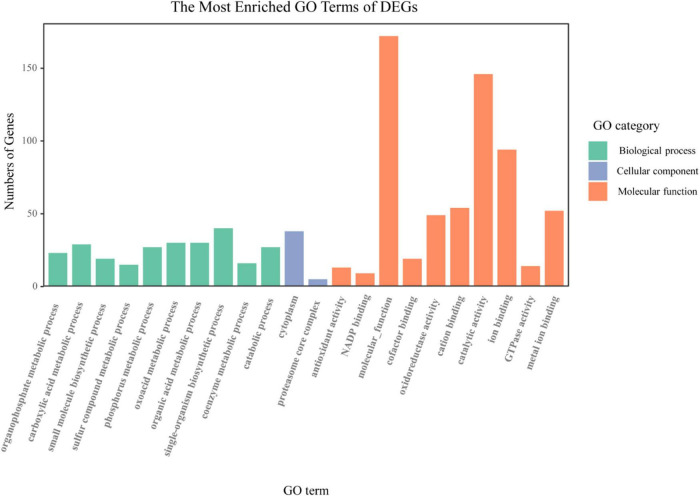
Gene ontology (GO) enrichment analysis of the AM-induced genes regulated by indigenous microorganisms. The *x*-axis indicates various GO terms. The *y*-axis indicates the number of genes in GO terms. The color of column indicates the GO category to which GO term belongs.

All the AM-induced genes regulated by indigenous microorganisms were mapped into the GO database and subjected to enrichment analysis to understand the function of these genes. GO enrichment analysis showed that 177 of 819 AM-induced genes regulated by indigenous microorganisms were significantly assigned (*q* < 0.05) to at least one GO term of the three major functional categories (Figure 3). In the BP class, “organophosphate metabolic process” (23 DEGs), “small molecule biosynthetic process” (19 DEGs), and “phosphorus metabolic process” (27 DEGs) were represented as marked; in the MF class, 13, 9, and 49 DEGs were identified as belonging to “antioxidant activity,” “NADP binding,” and “oxidoreductase activity,” respectively; in the CC class, 5 and 38 DEGs were identified as belonging to “cytoplasm” and “proteasome core complex,” respectively.

### Kyoto Encyclopedia of Genes and Genomes Pathway Enrichment Analysis of Arbuscular Mycorrhizal-Induced Genes Regulated by Indigenous Microorganisms

**FIGURE 4:**
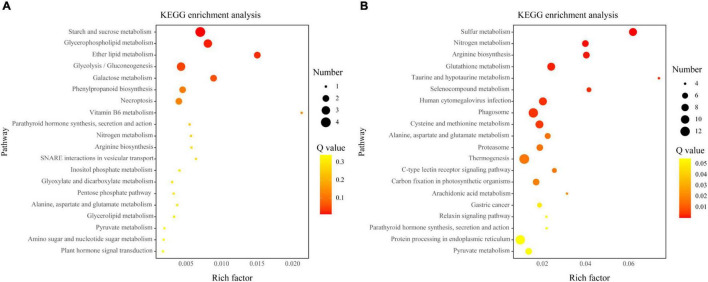
The Kyoto Encyclopedia of Genes and Genomes (KEGG) pathway enrichment analyses of the AM-induced genes upregulated **(A)** or downregulated **(B)** by indigenous microorganisms. The *x*-axis indicates the rich factors of pathways. The *y*-axis indicates various KEGG pathways. The size of the bubble indicates the number of genes enriched in this pathway. The color of the bubble indicates the significant degree of enrichment.

The KEGG pathway enrichment analysis identified a significant (*q* < 0.05) enrichment of 190 of 819 AM-induced genes regulated by indigenous microorganisms that involved in 24 pathways. In addition, compared to the *AMF* treatment, 125 AM-induced genes were upregulated under the *AMI* treatment, which has been enriched to “Carbohydrate metabolism” and “Lipid metabolism.” Conversely, 694 AM-induced genes were suppressed under the *AMI* treatment, and “Sulfur metabolism,” “Nitrogen metabolism,” and “Arginine biosynthesis,” which belong to energy metabolism and amino acid metabolism, were the most significant pathways that were enriched (Figure 4).

### Arbuscular Mycorrhizal-Induced Genes Regulated by Indigenous Microorganisms Associated With P, N, and Amino Acid Metabolisms

**FIGURE 5:**
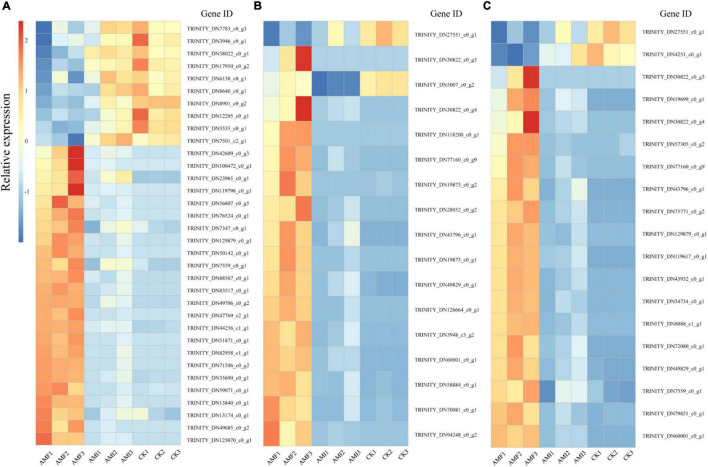
AM-induced genes regulated by indigenous microorganisms related to different categories. **(A)** AM-induced genes regulated by indigenous microorganisms related to P metabolism. **(B)** AM-induced genes regulated by indigenous microorganisms related to N metabolism. **(C)** AM-induced genes regulated by indigenous microorganisms related to amino acid metabolism. The horizontal axis represents the sample and the vertical axis represents the gene ID. The *AMF*, *AMI*, and *CK* mean the same as in Figure 1. Description and expression of genes related to different categories in *AMF* and *AMI* are listed in Supplementary Tables 3–5, respectively.

According to the results of enrichment analysis, AM-induced genes regulated by indigenous microorganisms associated with P, N, and amino acid metabolisms were selected for subsequent analysis (Figures 5A–C). There were 34 AM-induced genes regulated by indigenous microorganisms genes associated with P metabolism and 24 of which were downregulated by indigenous microorganisms (Figure 5A and Supplementary Table 3). Five genes (i.e., TRINITY_DN13840_c0_g1, TRINITY_DN100472_c0_g1, TRINITY_DN82958_c1_g1, TRINITY_DN7501_c2_g1, and TRINITY_DN38022_c0_g1) encoding Pi transporter (PT) were identified as DEGs associated with P transport. Particularly, the expression levels of TRINITY_DN13840_c0_g1, TRINITY_DN100472_c0_g1, and TRINITY_DN82958_c1_g1 were significantly lower under the *AMI* treatment compared to the *AMF* treatment and were downregulated by 27-, 20-, and 12-folds, respectively. In contrast, two DEGs (i.e., TRINITY_DN5555_c0_g1 and TRINITY_DN3946_c0_g1) related to the cellular response to Pi starvation were increased by 4- and 2-folds, respectively, under the *AMI* treatment. This mode of expression indicated that indigenous microorganisms significantly downregulated the expression of AM-induced genes related to P metabolism, especially the genes encoded for P transporters; however, they induced cellular response to Pi starvation.

The AM-induced genes regulated by indigenous microorganisms associated with N metabolism were also further analyzed, 16 of which were downregulated by indigenous microorganisms (Figure 5B and Supplementary Table 4). Particularly, a unigene (i.e., TRINITY_DN43796_c0_g1) encoding glutamine synthetase (GS) was significantly lower under the *AMI* treatment compared to the *AMF* treatment and was downregulated by 10 times. In addition, three clusters (i.e., TRINITY_DN77160_c0_g9, TRINITY_DN30822_c0_g4, and TRINITY_DN30822_c0_g3) encoding NAD-specific glutamate dehydrogenase 2-like (GDH2) were upregulated by AM fungi inoculation while downregulated by indigenous microorganisms. Furthermore, 2 unigenes (i.e., TRINITY_DN60001_c0_g1 and TRINITY_DN126664_c0_g1) encoding urease and urease accessory protein were regulated jointly by AM fungi and indigenous microorganisms in a similar pattern of GDH2. It can be concluded that the indigenous microorganisms significantly downregulated the expression of AM-induced genes encoding GS, GDH2, urease, and urease accessory protein, thereby offsetting the AM-induced benefits on N metabolism.

The 19 downregulation of AM-induced genes regulated by indigenous microorganisms involved in amino acid metabolism were observed (Figure 5C and Supplementary Table 5), including 6 clusters (i.e., TRINITY_DN60001_c0_g1, TRINITY_DN49829_c0_g1, TRINITY_DN77160_c0_g9, TRINITY_DN129879_c0_g1, TRINITY_DN30822_c0_g4, and TRINITY_DN30822_c0_g3) related to arginine biosynthesis, which were downregulated under the *AMI* and *CK* treatments. Meanwhile, 7 clusters involved in cysteine and methionine metabolism were detected, and the expression of 6 clusters (i.e., TRINITY_DN79851_c0_g1, TRINITY_DN43932_c0_g1, TRINITY_DN72080_c0_g1, TRINITY_DN75771_c0_g2, TRINITY_DN57305_c0_g2, and TRINITY_DN7559_c0_g1) was downregulated in the *AMI* and *CK* treatments, while one cluster (i.e., TRINITY_DN4251_c0_g1) with the extra function of ethylene synthesis in plant to resist pathogen infection was upregulated. Furthermore, a cluster related to alanine, aspartate, and glutamate metabolisms was also downregulated in the *AMI* and *CK* treatments. This mode of expression indicated that AM fungi could induce the synthesis and metabolism of amino acid by upregulated corresponding genes, while the indigenous microorganisms significantly offset this regulation.

## Discussion

### Indigenous Microorganisms Offset Benefits of Arbuscular Mycorrhizal Fungi for Host Plant on Growth and Nutrients of N and P

In this study, AM fungi inoculation differentially increased the biomass accumulation and nutrient utilization of N and P for the *B. tripartita* regardless of the presence or absence of indigenous microorganism (Table 1 and Figures 1A,B), which is consistent with H1 that AM fungi can promote the growth and the nutrient acquisition for *B. tripartita*. Meanwhile, previous studies have demonstrated the positive effect of AM fungi on the accumulation of mineral nutrients and the growth of host plants (Lin et al., 2017; Wang et al., 2017). He et al. (2007) have proven the benefits of AM fungi in the accumulation of biomass, N, and P for host plants cultivated in the karst soil. AM fungi have an important ecological function in promoting the growth and nutrient accumulation of host plants, which is mainly achieved through two pathways. First, AM fungi facilitate plants to forage nutrients directly (Smith and Read, 2008). For instance, AM fungi can absorb inorganic minerals from the soil and transfer them to host plants *via* hyphae (Wei et al., 2016). In addition, AM fungi could assist plants to expand their nutrient absorption area *via* hyphae and thus pass through the P depletion zone around the rhizosphere, which is formed due to the low fluidity and solubility of phosphorus (Bucher, 2007). Second, AM fungi can secrete soil enzymes, such as urease, leucine aminopeptidase, and alkaline phosphatase to mineralize the organic nutrients and dissociate the insoluble nutrients, thereby increasing plant nutrient absorption to promote plant growth (Burke et al., 2011; Li et al., 2017; Liu et al., 2021). Furthermore, AM fungi significantly decreased the N/P ratio of *B. tripartita* from 26.38 to 16.53 by increasing the accumulation of P in this experiment (Figure 1C), which indicated that AM fungi are more effective in assisting plants to obtain P than N and native plant *B. tripartita* may be limited by P in karst areas without AM fungi, according to Koerselman and Meuleman (1996) who reported that plants with an N/P ratio greater than 16 are thought to be mainly limited in growth by P. This result is consistent with previous research which showed that AM fungi reduced N/P ratio of the host plant by promoting P absorption, resulting in P no longer being the main limiting factor of plant growth (Shen et al., 2020). However, the AM-induced benefit in biomass accumulation and nutrient utilization of N and P was offset by the presence of indigenous microorganisms (Table 1 and Figures 1A,B). These offsets could verify H1 that indigenous microorganisms can offset the benefits of AM fungi. Consistent with our results, the offset on AM-induced benefit had been demonstrated in grapevine inoculated by a mixed microbial inoculum when compared to a single AM fungi inoculum (Balestrini et al., 2016). A meta-analysis revealed that AM-induced benefit in biomass accumulation and nutrient utilization is generally correlated with mycorrhizal colonization (Treseder, 2013). Conversely, the mycorrhizal colonization of *B. tripartita* was not significantly decreased by the indigenous microorganisms (Table 1). In addition, this study also finds the expression of genes that had been proved to be involved in the evolutionary conservative processes supporting symbioses (Guether et al., 2009), such as chitinases (14 unigenes), Ser/Thr receptor kinases (90 unigenes), glutathione *S*-transferases (22 unigenes), and lectins (37 unigenes) in mycorrhizal roots under *AMF* and *AMI* treatments, and 126 of 144 genes were not identified as DEGs between treatments with or without indigenous microorganisms (Supplementary Table 6). The abovementioned morphological and transcriptome evidence suggested that indigenous microorganisms did not offset the AM-induced benefits by reducing the mycorrhizal colonization but may offset by downregulating nutrient metabolism processes due to the co-regulation on nutrient metabolism by AM fungi and other microorganisms (Ruzicka et al., 2011; Nanjareddy et al., 2017; Salas-Gonzalez et al., 2021).

### Indigenous Microorganisms Downregulated the Arbuscular Mycorrhizal-Induced Genes Related to P Metabolism

In this study, GO enrichment analysis suggested that the offset of indigenous microorganisms for AM-induced benefits on P absorption was related to the regulation of indigenous microorganisms on AM-induced genes associated with P metabolism (Figure 3 and Supplementary Table 3), which verified the H2 that indigenous microorganisms negatively modulate AM-induced nutrient metabolism genes to offset the benefits of AM fungi on plant growth and nutrient accumulation. Subsequently, the expression and functions of these unigenes were analyzed in depth. Mycorrhizal plants have two potential pathways for P absorption, one is a direct pathway by root epidermal cells and root hairs which is mediated by the activities of a PT of the Pht1 family, and another is the mycorrhizal pathway *via* the AM fungal symbiont, which is depended on the activities of PHO84, PHO89, PHO91, and mycorrhiza-inducible PT (MPT) (Ferrol et al., 2019). In the current transcriptome, the unigenes homologous to the genes encoding the PT and MPT was identified (Supplementary Table 3). TRINITY_DN13840_c0_g1 and TRINITY_DN100472_c0_g1 encoding PT PHO1 and its homologs, which involved the transportation of Pi from the root to the shoot (Khan et al., 2014), were upregulated by AM fungi. Consistently, the positive modulation of AM fungi inoculation on PHO1 was observed by Abbaspour et al. (2021), which indicated the ability of AM fungi to take up P at a lower threshold due to the expression of high-affinity PT genes (Selvaraj and Chellappan, 2006). The expression of PHO1 was downregulated by indigenous microorganisms, indicating that the suppression of AM-induced benefits of P uptake may owe to the negative modulation of indigenous microorganisms on PT. In addition, TRINITY_DN38022_c0_g1 and TRINITY_DN7501_c2_g1 encoding PHO84 were detected in mycorrhizal roots without indigenous microorganisms, and these genes are typical mycorrhiza-induced transporters according to previous studies. Mycorrhiza-induced transporters homologous to PHO84 were identified in *Solanum tuberosum* L. (Rausch et al., 2001), *M. truncatula* (Harrison et al., 2002), and other plant species, such as *Lycopersicon esculentum* M., *Sorghum bicolor*, and *Linum usitatissimum* L. (Poulsen et al., 2005; Walder et al., 2015). Interestingly, genes encoding PHO84 had a significantly higher expression in mycorrhizal roots with indigenous microorganisms and non-mycorrhizal roots (Supplementary Table 3), which is consistent with the results of Volpe et al. (2016). This phenomenon may have a positive effect on the adaptation of *B. tripartita* to the lack of soil P content and the offset of indigenous microorganisms in P uptake since PHO84 can regulate the response of root branching to Pi deficiency (Volpe et al., 2016). In addition, the higher expression of PHO84 genes and its homologs in mycorrhizal roots with indigenous microorganisms and non-mycorrhizal roots may relate to their additional roles as the Pi-starvation marker genes and transceptors in the non-mycorrhizal Pi uptake pathway that act upstream of the Pi-sensing machinery (Popova et al., 2010). The expression of PHO84 in root tissue with lower Pi content, which involves Pi sensing, may be an adaptive mechanism of karst adaptive plants to Pi deficiency. Overall, AM fungi can upregulate the expression of high-affinity PTs, thereby increasing P absorption and transport in plants, while indigenous microorganisms downregulate the expression of these genes, thereby offset the AM-induced benefits on P metabolism. Interestingly, a special PT is highly expressed under the offset of indigenous microorganisms and non-mycorrhizal, which may be an adaptation mechanism of karst adaptive plants to P deficiency.

### Indigenous Microorganisms Downregulated the Arbuscular Mycorrhizal-Induced Genes Related to N Metabolism

In this study, the KEGG enrichment analysis suggested that N metabolism was induced by AM inoculation while regulated by indigenous microorganisms (Figure 4), which also verified the H2. The plants have developed a complex N metabolism system to utilize essential N for synthesizing protein and other nitrogenous compounds, which includes N acquisition, transport, and assimilation (Qu et al., 2020). In addition, the regulation and mechanism of AM fungi on plant N metabolism were elucidated by Ruzicka et al. (2011). For example, AM fungi were reported to regulate the assimilation of N in host roots through increasing the activity and accumulation of GS, GDH, and asparagine synthase under low external N concentrations (Subramanian and Charest, 1998; Ruzicka et al., 2011). In this study, 16 of 17 AM-induced genes regulated by indigenous microorganisms associated with N metabolism were upregulated by AM fungi inoculation while downregulated by indigenous microorganisms (Supplementary Table 4 and Figure 5B). In detail, the glnA (TRINITY_DN43796_c0_g1) encoding GS, and thus serves for primary assimilation of N (Zhao and Shi, 2006), was significantly upregulated by AM fungi, and in the consistent experiment, the increased activity of glnA in mycorrhizal roots of *Daucus carota* L. was observed (Toussaint et al., 2004). In addition, the DEGs described as GDH2 encoding NAD-specific GDH-like genes (TRINITY_DN77160_c0_g9, TRINITY_DN30822_c0_g4, and TRINITY_DN30822_c0_g3) were upregulated by AM fungi, and the higher expression of GDH2 in mycorrhizal roots was also observed by Toussaint et al. (2004). The higher expression of the abovementioned genes can more effectively convert glutamate into 2-oxoglutarate with NADH generation. Conversely, 2-oxoglutarate can also more effectively convert back to glutamate by the NADPH-dependent GDH with the consumption of NADPH (Boles et al., 1993). This efficient conversion can promote N assimilation and amino acid metabolism (Qu et al., 2020). This differential expression indicated that AM fungi promoted the N metabolism by upregulated the expressions of glnA and GDH2, which are the key enzymes of N assimilation; however, the indigenous microorganism downregulated this expression, thereby inhibiting the AM-induced benefits on N metabolism. Subsequently, AM-induced genes regulated by indigenous microorganisms related to the arginine biosynthesis, which is a downstream pathway of N metabolism, were analyzed emphatically. In the present transcriptome, the abovementioned three clusters described as GDH2 and a cluster described as glnA were also identified to participate in the arginine biosynthesis by regulating the interconversion between glutamate and NH_3_ to provide the precursor for arginine biosynthesis and urea cycle (Winter et al., 2015). In addition, transcriptome analysis revealed that genes encoding urease and urease accessory protein (TRINITY_DN60001_c0_g1 and TRINITY_DN126664_c0_g1) were also regulated by AM fungi jointly with indigenous microorganisms in a consistent pattern with glnA and GDH2, which involved in N recycling from ureide, purine, and arginine catabolism in plants (Witte et al., 2005). Overall, the change of transcriptome reveals that indigenous microorganisms downregulated the AM-induced genes encoding key enzymes of N assimilation and urea cycle, thereby achieving the negative modulations on N metabolism and amino acid metabolism in plant roots.

## Conclusion

The AM fungi significantly enhanced the plant biomass, N, and P accumulation with the reduction of plants’ N/P ratio, while the indigenous microorganisms offset the AM-induced benefits on plant growth and nutrient acquisition. Subsequently, 819 AM-induced genes regulated by indigenous microorganisms were identified in this experiment. The enrichment analysis suggested that these genes were highly associated with the metabolic processes of organophosphate, P, and N, which was consistent with the plant nutrients concentration in this experiment. A total of 34 DEGs were related to P and 17 for N metabolic pathways. Especially, compared to AM fungi inoculation, two unigenes (i.e., TRINITY_DN38022_c0_g1 and TRINITY_DN7501_c2_g1) encoding PHO1 were downregulated by 27- and 20-folds, respectively, under AM fungi combining with indigenous microorganisms; the expression of unigene (TRINITY_DN43796_c0_g1) encoding GS was downregulated by 10-fold; the expressions of three unigenes (i.e., TRINITY_DN77160_c0_g9, TRINITY_DN30822_c0_g4, and TRINITY_DN30822_c0_g3) encoding GDH2 were downregulated by 12-, 20-, and 100-folds, respectively; 2 unigenes (i.e., TRINITY_DN60001_c0_g1 and TRINITY_DN126664_c0_g1) encoding urease and urease accessory protein were downregulated by 12- and 20-folds. In conclusion, we suggested that AM fungi can promote plant growth and nutrient absorption, while indigenous microorganisms offset the AM-induced benefits on plant growth and nutrient acquisition through regulating the genes related to P transport and N assimilation.

## Data Availability Statement

The data presented in the study are deposited in the NCBI SRA repository, accession number PRJNA817834.

## Author Contributions

YH designed the experiments. XH conducted the experiment. WR carried out the bioinformatics analyses and writing. WR, YG, YS, and QL analyzed the data. WR, PW, BW, TX, and KS revised and proofread the manuscript. All authors contributed to the article and approved the submitted version.

## Conflict of Interest

The authors declare that the research was conducted in the absence of any commercial or financial relationships that could be construed as a potential conflict of interest.

## Publisher’s Note

All claims expressed in this article are solely those of the authors and do not necessarily represent those of their affiliated organizations, or those of the publisher, the editors and the reviewers. Any product that may be evaluated in this article, or claim that may be made by its manufacturer, is not guaranteed or endorsed by the publisher.

## Footnotes

1
https://github.com/trinityrnaseq/trinityrnaseq/wiki

2
http://busco.ezlab.org

3
https://github.com/tanghaibao/goatools/

4
www.majorbio.com

5
http://kobas.cbi.pku.edu.cn/home.do
