# Supplementary material for: Indigenous Microorganisms Offset Arbuscular Mycorrhizal Fungi-Induced Plant Growth and Nutrient Acquisition Through Negatively Modulating the Genes of Phosphorus Transport and Nitrogen Assimilation
Source: Front Plant Sci. 2022 May 9;13:880181. doi: 10.3389/fpls.2022.880181 (PMC9125159; doi:10.3389/fpls.2022.880181)
Supplement: Supplementary file 1 [file Data_Sheet_1.docx]

Supplementary Material

# Supplementary Figures and Tables

- 1. **Supplementary Figures**

| **Tabel S1 The distribution of sequence length** | | |
| --- | --- | --- |
| **Length** | **Number of unigenes** | **Percent of unigenes** |
| 0~500 | 64069 | 50% |
| 501~1000 | 31271 | 24% |
| 1001~1500 | 13016 | 10% |
| 1501~2000 | 8334 | 7% |
| 2001~2500 | 4772 | 4% |
| 2501~3000 | 2688 | 2% |
| 3001~3500 | 1485 | 1% |
| 3501~4000 | 836 | 1% |
| 4001~4500 | 470 | 0% |
| >4500 | 758 | 1% |

| **Table S2 Detail of Transcriptome annotation** | | | | |
| --- | --- | --- | --- | --- |
| **database** | **Exp Unigene  number (percent)** | **Exp Transcript  number (percent)** | **All Unigene  number (percent)** | **All Transcript  number (percent)** |
|  |  |  |  |  |
|  |  |  |  |  |
| GO | 52318(41.78%) | 94756(48.27%) | 52897(41.42%） | 95652(47.89%） |
| KEGG | 35876(28.65%) | 59350(30.23%) | 36368(28.48%） | 60040(30.12%） |
| COG | 59601(47.59%) | 105270(53.63%) | 60268(47.20%） | 106276(53.31%） |
| NR | 64174(52.25%) | 115488(58.83%） | 64915(50.83%） | 116607(58.49%） |
| Swiss-Prot | 53914(43.05%) | 93133(47.44%） | 54516(42.69%） | 94045(47.18%） |
| Pfam | 55025(43.94%) | 93126(47.44%） | 55577(43.52%） | 93976(47.14%） |
| Total annotation | 74394(59.41%) | 127316(64.84%） | 75348(59.00%） | 128659(64.51%） |
| GO, Gene Ontology; KEGG, KEGG Ontology database; COG, Clusters of Orthologous Groups of Proteins; NR, non-redundant protein sequences database. | | | | |

| **Table S3 AM-induced genes regulated by indigenous microorganisms related to P metabolism** | | | | | | | |
| --- | --- | --- | --- | --- | --- | --- | --- |
| **Gene name** | **Gene description** | ***AMI*** | ***AMF*** | **Log_2_*Fold Change*** | ***q*-value** | **Regulation** | **KO name** |
| TRINITY_DN47769_c2_g1 | 3'(2'),5'-bisphosphate nucleotidase-like | 1.90 | 0.02 | -6.18 | 0.000 | down | cysQ, MET22, BPNT1 |
| TRINITY_DN49685_c0_g2 | alpha,alpha-trehalose-phosphate synthase -like | 1.25 | 0.02 | -5.49 | 0.001 | down | otsA |
| TRINITY_DN51871_c0_g1 | molybdenum cofactor sulfurase isoform X1 | 3.02 | 0.06 | -5.41 | 0.000 | down | ABA3 |
| TRINITY_DN59071_c0_g1 | farnesyl pyrophosphate synthase-like | 1.88 | 0.04 | -5.37 | 0.000 | down | FDPS |
| TRINITY_DN50142_c0_g1 | hypothetical protein BHE74_00000187 | 1.45 | 0.03 | -5.14 | 0.001 | down | E2.2.1.2, talA, talB |
| TRINITY_DN119798_c0_g1 | alpha,alpha-trehalose-phosphate synthase -like | 2.31 | 0.07 | -4.83 | 0.000 | down | otsA |
| TRINITY_DN13840_c0_g1 | phosphate transporter PHO1 | 4.98 | 0.12 | -4.77 | 0.000 | down | ------ |
| TRINITY_DN88567_c0_g1 | nucleotide diphosphate kinase 1 | 6.69 | 0.24 | -4.49 | 0.000 | down | ndk, NME |
| TRINITY_DN125070_c0_g1 | ribulose-phosphate 3-epimerase, cytoplasmic isoform isoform X2 | 1.66 | 0.07 | -4.42 | 0.003 | down | rpe, RPE |
| TRINITY_DN100472_c0_g1 | phosphate transporter PHO1 homolog 10 isoform X1 | 0.96 | 0.04 | -4.33 | 0.004 | down | ------ |
| TRINITY_DN71506_c0_g2 | glucose-6-phosphate 1-dehydrogenase | 1.04 | 0.04 | -4.29 | 0.003 | down | G6PD, zwf |
| TRINITY_DN76524_c0_g1 | probable aminotransferase sirI | 0.91 | 0.04 | -4.18 | 0.000 | down | ------ |
| TRINITY_DN83517_c0_g1 | 6-phosphogluconate dehydrogenase, decarboxylating 1-like | 2.50 | 0.13 | -4.03 | 0.000 | down | PGD, gnd, gntZ |
| TRINITY_DN56807_c0_g5 | RecName: Full=Probable pyridoxal 5'-phosphate synthase subunit PDX1; | 1.23 | 0.06 | -3.98 | 0.009 | down | pdxS, pdx1 |
| TRINITY_DN129879_c0_g1 | PREDICTED: alanine aminotransferase 2-like | 1.50 | 0.08 | -3.93 | 0.000 | down | GPT, ALT |
| TRINITY_DN35690_c0_g1 | fructose-bisphosphate aldolase 1-like | 5.90 | 0.35 | -3.80 | 0.000 | down | FBA, fbaA |
| TRINITY_DN82958_c1_g1 | probable mitochondrial phosphate carrier protein | 1.28 | 0.08 | -3.59 | 0.001 | down | SLC25A3, PHC, PIC |
| TRINITY_DN23965_c0_g1 | hypothetical protein Zm00014a_043352 | 7.75 | 1.91 | -3.58 | 0.000 | down | ABA3 |
| TRINITY_DN42609_c0_g3 | predicted protein | 1.96 | 0.17 | -3.21 | 0.008 | down | pdxS, pdx1 |
| TRINITY_DN49706_c0_g2 | inositol-3-phosphate synthase-like | 1.49 | 0.15 | -3.12 | 0.000 | down | INO1, ISYNA1 |
| TRINITY_DN44256_c1_g1 | unknown | 2.91 | 0.31 | -2.87 | 0.000 | down | GAPDH, gapA |
| TRINITY_DN7559_c0_g1 | tyrosine aminotransferase-like | 36.29 | 4.95 | -2.70 | 0.001 | down | TAT |
| TRINITY_DN13174_c0_g1 | protein NUCLEAR FUSION DEFECTIVE 4-like | 5.37 | 1.47 | -1.54 | 0.001 | down | ------ |
| TRINITY_DN7347_c0_g1 | type I inositol polyphosphate 5-phosphatase 8 isoform X1 | 10.18 | 3.62 | -1.18 | 0.002 | down | ------ |
| TRINITY_DN3946_c0_g1 | SPX domain-containing protein 1-like | 74.43 | 108.94 | 1.13 | 0.002 | up | ------ |
| TRINITY_DN6138_c0_g1 | glucose-1-phosphate adenylyltransferase small subunit, chloroplastic/amyloplastic | 13.74 | 22.87 | 1.14 | 0.001 | up | glgC |
| TRINITY_DN12205_c0_g1 | non-specific phospholipase C1 | 6.67 | 11.68 | 1.15 | 0.000 | up | plc |
| TRINITY_DN7501_c2_g1 | putative phosphate transporter 1,7 | 5.51 | 9.75 | 1.21 | 0.000 | up | PHO84 |
| TRINITY_DN8640_c0_g1 | UTP--glucose-1-phosphate uridylyltransferase 3, chloroplastic | 15.71 | 31.45 | 1.36 | 0.000 | up | ------ |
| TRINITY_DN7783_c0_g3 | hypothetical protein LSAT_5X5301 | 2.28 | 4.56 | 1.38 | 0.001 | up | SLC35E1 |
| TRINITY_DN17950_c0_g2 | NADP-dependent glyceraldehyde-3-phosphate dehydrogenase-like | 5.98 | 16.08 | 1.92 | 0.000 | up | gapN |
| TRINITY_DN5555_c0_g1 | SPX domain-containing protein 3-like | 13.18 | 49.40 | 2.06 | 0.001 | up | ------ |
| TRINITY_DN8901_c0_g2 | glyceraldehyde-3-phosphate dehydrogenase, cytosolic | 1.87 | 15.15 | 3.30 | 0.000 | up | GAPDH, gapA |
| TRINITY_DN38022_c0_g1 | probable inorganic phosphate transporter 1-7 | 0.76 | 8.40 | 3.90 | 0.001 | up | PHO84 |
| *Fold Change*, the ratio of expressions in *AMI* and *AMF* treatment of same gene; *q*-value, *p*-value adjusted by Benjamini and Hochberg method. | | | | | | | |

| **Table S4 AM-induced genes regulated by indigenous microorganisms related to N metabolism** | | | | | | | | |
| --- | --- | --- | --- | --- | --- | --- | --- | --- |
| **Gene name** | **Gene description** | | ***AMI*** | ***AMF*** | **Log_2_*Fold Change*** | ***q*-value** | **Regulation** | **KO name** |
| TRINITY_DN118200_c0_g1 | hypothetical protein AXX17_ATUG04840 | 0.00 | | 2.32 | -7.39 | 0.0000 | down | ------ |
| TRINITY_DN19873_c0_g2 | unknown | 0.00 | | 1.50 | -6.85 | 0.0000 | down | ------ |
| TRINITY_DN30822_c0_g3 | NAD-specific glutamate dehydrogenase-like | 0.00 | | 0.71 | -6.61 | 0.0046 | down | GDH2 |
| TRINITY_DN94248_c0_g2 | PREDICTED: alkaline ceramidase 3-like | 0.04 | | 2.21 | -5.47 | 0.0000 | down | ACER3, YDC1 |
| TRINITY_DN19873_c0_g1 | unknown | 0.07 | | 3.19 | -5.46 | 0.0000 | down | ------ |
| TRINITY_DN70081_c0_g1 | hypothetical protein F511_08091 | 0.17 | | 4.19 | -4.49 | 0.0018 | down | cynT, can |
| TRINITY_DN5007_c0_g2 | carbonic anhydrase-like isoform X1 | 0.34 | | 8.74 | -4.46 | 0.0000 | down | cynT, can |
| TRINITY_DN126664_c0_g1 | uncharacterized urease accessory protein ureG-like | 0.07 | | 1.52 | -4.19 | 0.0006 | down | ureG |
| TRINITY_DN77160_c0_g9 | NAD-specific glutamate dehydrogenase-like | 0.09 | | 1.94 | -4.13 | 0.0002 | down | GDH2 |
| TRINITY_DN58884_c0_g1 | unknown | 0.25 | | 4.20 | -4.03 | 0.0000 | down | ------ |
| TRINITY_DN30822_c0_g4 | NAD-specific glutamate dehydrogenase-like | 0.14 | | 2.44 | -3.91 | 0.0008 | down | GDH2 |
| TRINITY_DN28052_c0_g2 | unknown | 0.11 | | 1.99 | -3.89 | 0.0008 | down | cah |
| TRINITY_DN3948_c3_g2 | nitrogen network kinase 1-like | 0.17 | | 2.15 | -3.45 | 0.0049 | down | ------ |
| TRINITY_DN60001_c0_g1 | PREDICTED: urease isoform X1 | 0.13 | | 1.52 | -3.22 | 0.0000 | down | ureC;URE |
| TRINITY_DN43796_c0_g1 | glutamine synthetase-like | 0.58 | | 5.85 | -3.12 | 0.0073 | down | glnA, GLUL |
| TRINITY_DN49829_c0_g1 | hypothetical protein F511_09367 | 0.25 | | 1.96 | -2.66 | 0.0000 | down | E3.5.3.1, rocF, arg |
| TRINITY_DN27551_c0_g1 | putative glutamine synthetase | 1.59 | | 0.71 | 1.73 | 0.0019 | up | glnA, GLUL |
| *Fold Change*, the ratio of expressions in *AMI* and *AMF* treatment of same gene; *q*-value, *p*-value adjusted by Benjamini and Hochberg method. | | | | | | | | |

| **Table S5 AM-induced genes regulated by indigenous microorganisms related to amimo acid metabolism** | | | | | | | | |
| --- | --- | --- | --- | --- | --- | --- | --- | --- |
| **Gene name** | **Gene description** | ***AMI*** | ***AMF*** | **Log_2_*Fold Change*** | ***q*-value** | | **Regulation** | **KO name** |
| TRINITY_DN30822_c0_g3 | NAD-specific glutamate dehydrogenase-like | 0.00 | 0.71 | -6.61 | | 0.0046 | down | GDH2 |
| TRINITY_DN57305_c0_g2 | glutathione synthetase, chloroplastic-like isoform X1 | 0.02 | 1.02 | -5.62 | | 0.0006 | down | GSS |
| TRINITY_DN8088_c1_g1 | delta-1-pyrroline-5-carboxylate dehydrogenase, mitochondrial-like | 0.10 | 2.17 | -4.21 | | 0.0000 | down | E1.2.1.88 |
| TRINITY_DN77160_c0_g9 | NAD-specific glutamate dehydrogenase-like | 0.09 | 1.94 | -4.13 | | 0.0002 | down | GDH2 |
| TRINITY_DN129879_c0_g1 | PREDICTED: alanine aminotransferase 2-like | 0.08 | 1.50 | -3.93 | | 0.0002 | down | GPT, ALT |
| TRINITY_DN30822_c0_g4 | NAD-specific glutamate dehydrogenase-like | 0.14 | 2.44 | -3.91 | | 0.0008 | down | GDH2 |
| TRINITY_DN43932_c0_g1 | S-adenosylmethionine synthase-like | 0.12 | 1.98 | -3.82 | | 0.0000 | down | metK |
| TRINITY_DN34734_c0_g1 | malate dehydrogenase, cytoplasmic-like | 0.09 | 1.34 | -3.61 | | 0.0001 | down | MDH2 |
| TRINITY_DN79851_c0_g1 | uncharacterized protein LOC111998198 | 0.12 | 1.66 | -3.47 | | 0.0000 | down | mmuM, BHMT2 |
| TRINITY_DN60001_c0_g1 | PREDICTED: urease isoform X1 | 0.13 | 1.52 | -3.22 | | 0.0000 | down | ureC;URE |
| TRINITY_DN75771_c0_g2 | uncharacterized protein LOC106780750, partial | 0.21 | 2.30 | -3.17 | | 0.0000 | down | metE |
| TRINITY_DN43796_c0_g1 | glutamine synthetase-like | 0.58 | 5.85 | -3.12 | | 0.0073 | down | glnA, GLUL |
| TRINITY_DN19699_c0_g1 | PREDICTED: L-lactate dehydrogenase B-like | 0.53 | 4.24 | -2.87 | | 0.0042 | down | LDH, ldh |
| TRINITY_DN7559_c0_g1 | tyrosine aminotransferase-like | 4.95 | 36.29 | -2.70 | | 0.0010 | down | TAT |
| TRINITY_DN49829_c0_g1 | hypothetical protein F511_09367 | 0.25 | 1.96 | -2.66 | | 0.0000 | down | E3.5.3.1, rocF, arg |
| TRINITY_DN72080_c0_g1 | unknown | 0.25 | 1.87 | -2.56 | | 0.0001 | down | E3.3.1.1, ahcY |
| TRINITY_DN119617_c0_g1 | malate dehydrogenase, mitochondrial-like | 0.14 | 0.89 | -2.35 | | 0.0062 | down | MDH2 |
| TRINITY_DN4251_c0_g1 | 1-aminocyclopropane-1-carboxylate oxidase-like | 40.15 | 16.60 | 1.64 | | 0.0002 | up | E1.14.17.4 |
| TRINITY_DN27551_c0_g1 | putative glutamine synthetase | 1.59 | 0.71 | 1.73 | | 0.0019 | up | glnA, GLUL |
| *Fold Change*, the ratio of expressions in *AMI* and *AMF* treatment of same gene; *q*-value, *p*-value adjusted by Benjamini and Hochberg method. | | | | | | | | |

| **Table S6 The unigenes related to the evolutionary conservation processes supporting symbiosis** | | | | | | | |
| --- | --- | --- | --- | --- | --- | --- | --- |
| **Gene name** | **Gene description** | ***AMI*** | ***AMF*** | **Log_2_*Fold Change*** | ***q*-value** | **Regulation** | **KO name** |
| TRINITY_DN131074_c0_g1 | PREDICTED: glutaredoxin-like | 1.05 | 16.09 | -3.72 | 0.0102 | not significant | grxC, GLRX, GLRX2 |
| TRINITY_DN77915_c0_g1 | kinase family protein | 0.04 | 0.89 | -4.17 | 0.0151 | not significant | ------ |
| TRINITY_DN77212_c0_g4 | 40S ribosomal protein S10-1 | 0.16 | 2.44 | -3.77 | 0.0154 | not significant | RP-S10e, RPS10 |
| TRINITY_DN2320_c1_g1 | serine/threonine-protein kinase STY46-like isoform X2 | 11.87 | 32.81 | -1.20 | 0.0227 | not significant | ------ |
| TRINITY_DN2957_c1_g1 | probable LRR receptor-like serine/threonine-protein kinase At1g06840 isoform X1 | 16.73 | 30.55 | -0.61 | 0.0427 | not significant | ------ |
| TRINITY_DN8941_c0_g1 | probable serine/threonine-protein kinase PBL17 | 10.95 | 24.29 | -0.79 | 0.0539 | not significant | IRAK4 |
| TRINITY_DN61708_c0_g3 | putative sterigmatocystin biosynthesis protein stcT | 0.19 | 0.97 | -2.18 | 0.0968 | not significant | EEF1G |
| TRINITY_DN25266_c0_g1 | CBL-interacting serine/threonine-protein kinase 25-like | 18.55 | 41.74 | -0.86 | 0.1243 | not significant | PRKAA, AMPK |
| TRINITY_DN19585_c0_g1 | Tyrosine kinase | 0.24 | 2.21 | -3.06 | 0.1563 | not significant | ANKRD17, MASK |
| TRINITY_DN15329_c0_g2 | CBL-interacting serine/threonine-protein kinase 5-like | 9.52 | 18.32 | -0.63 | 0.1621 | not significant | PRKAA, AMPK |
| TRINITY_DN46718_c0_g1 | cysteine-rich receptor-like protein kinase 19 isoform X2 | 0.27 | 1.51 | -2.30 | 0.1677 | not significant | IRAK4 |
| TRINITY_DN3921_c0_g1 | CBL-interacting serine/threonine-protein kinase 9-like isoform X1 | 24.81 | 37.24 | -0.47 | 0.1996 | not significant | ------ |
| TRINITY_DN47315_c0_g1 | serine/threonine-protein kinase HT1-like | 0.44 | 1.83 | -1.75 | 0.2212 | not significant | ------ |
| TRINITY_DN45835_c0_g5 | Carbon catabolite-derepressing protein kinase | 0.20 | 1.03 | -2.13 | 0.2236 | not significant | ------ |
| TRINITY_DN3651_c0_g2 | probable serine/threonine-protein kinase PBL3 | 6.94 | 17.78 | -1.09 | 0.2776 | not significant | IRAK4 |
| TRINITY_DN14083_c1_g2 | putative malectin | 2.09 | 3.61 | -0.47 | 0.3182 | not significant | ------ |
| TRINITY_DN54307_c0_g1 | putative sterigmatocystin biosynthesis protein stcT | 0.35 | 1.10 | -1.31 | 0.3392 | not significant | EEF1G |
| TRINITY_DN11298_c0_g1 | chitinase 2-like | 32.07 | 89.20 | -1.26 | 0.3604 | not significant | ------ |
| TRINITY_DN57538_c0_g1 | glutathione-s-transferase theta, gst, putative | 0.52 | 2.52 | -2.14 | 0.3726 | not significant | yghU, yfcG |
| TRINITY_DN61711_c0_g1 | 40S ribosomal protein S10-A-like | 0.67 | 2.21 | -1.46 | 0.3918 | not significant | RP-S10e, RPS10 |
| TRINITY_DN404_c0_g2 | glutathione S-transferase U17-like | 2.18 | 5.64 | -1.10 | 0.3934 | not significant | GST, gst |
| TRINITY_DN14329_c0_g1 | G-type lectin S-receptor-like serine/threonine-protein kinase LECRK2 | 0.55 | 1.54 | -1.19 | 0.3967 | not significant | ------ |
| TRINITY_DN25424_c0_g1 | hypothetical protein SEVIR_4G298400v2 | 0.96 | 3.28 | -1.53 | 0.4048 | not significant | ------ |
| TRINITY_DN67552_c0_g1 | L-type lectin-like domain-containing protein C126.08c | 0.15 | 0.66 | -1.97 | 0.4124 | not significant | LMAN1, ERGIC53 |
| TRINITY_DN36134_c0_g1 | putative leucine-rich repeat receptor-like serine/threonine-protein kinase At2g24130 | 1.03 | 2.56 | -1.09 | 0.4197 | not significant | ------ |
| TRINITY_DN77656_c0_g1 | serine/threonine-protein kinase hal4-like | 1.16 | 2.91 | -2.14 | 0.4618 | not significant | E2.7.11.- |
| TRINITY_DN19634_c0_g1 | putative aspartic peptidase A1 family | 1.28 | 3.37 | -1.20 | 0.4661 | not significant | ------ |
| TRINITY_DN8155_c0_g1 | putative serine-threonine/tyrosine-protein kinase catalytic domain-containing protein | 32.94 | 55.14 | -0.44 | 0.4804 | not significant | ------ |
| TRINITY_DN11229_c0_g2 | probable glutathione S-transferase parC | 4.54 | 10.72 | -0.72 | 0.4867 | not significant | GST, gst |
| TRINITY_DN8736_c0_g1 | putative serine/threonine-protein kinase isoform X1 | 2.67 | 4.49 | -0.43 | 0.5044 | not significant | IRAK4 |
| TRINITY_DN13967_c0_g2 | CBL-interacting serine/threonine-protein kinase 14-like | 2.53 | 4.71 | -0.63 | 0.5163 | not significant | SNF1 |
| TRINITY_DN31341_c0_g2 | probable L-type lectin-domain containing receptor kinase S.5 | 13.21 | 21.61 | -0.40 | 0.5345 | not significant | IRAK4 |
| TRINITY_DN35134_c0_g2 | probable receptor-like serine/threonine-protein kinase At5g57670 | 1.06 | 2.24 | -0.62 | 0.5465 | not significant | ------ |
| TRINITY_DN16568_c0_g1 | LRR receptor-like serine/threonine-protein kinase GSO1 | 4.34 | 6.98 | -0.38 | 0.5787 | not significant | ------ |
| TRINITY_DN46476_c0_g3 | serine/threonine-protein kinase SKM1-like | 0.33 | 0.86 | -1.00 | 0.5801 | not significant | CLA4 |
| TRINITY_DN7211_c0_g2 | probable receptor-like protein kinase At2g23200 | 1.92 | 3.07 | -0.36 | 0.5813 | not significant | ------ |
| TRINITY_DN87803_c0_g1 | mRNA cap guanine-N7 methyltransferase 1 | 0.73 | 2.10 | -1.42 | 0.6894 | not significant | GST, gst |
| TRINITY_DN29509_c0_g1 | serine/threonine-protein kinase ATG1c-like | 16.48 | 23.34 | -0.22 | 0.7195 | not significant | ULK2, ATG1 |
| TRINITY_DN15696_c0_g1 | elongation factor 1-gamma 2-like | 0.43 | 0.83 | -0.75 | 0.7263 | not significant | EEF1G |
| TRINITY_DN38785_c0_g1 | PREDICTED: glutathione S-transferase F13 isoform X1 | 1.31 | 3.48 | -1.28 | 0.7277 | not significant | GST, gst |
| TRINITY_DN21351_c0_g1 | G-type lectin S-receptor-like serine/threonine-protein kinase SD2-5 | 1.49 | 2.31 | -0.38 | 0.7435 | not significant | ------ |
| TRINITY_DN35074_c0_g1 | serine/threonine-protein kinase ENV7-like | 0.28 | 0.61 | -0.94 | 0.7617 | not significant | STK16 |
| TRINITY_DN6153_c0_g2 | Leucine-rich repeat-containing protein | 4.41 | 6.23 | -0.19 | 0.7719 | not significant | ------ |
| TRINITY_DN2097_c0_g1 | G-type lectin S-receptor-like serine/threonine-protein kinase At4g27290 | 5.40 | 7.89 | -0.27 | 0.7734 | not significant | IRAK4 |
| TRINITY_DN4494_c0_g1 | CBL-interacting serine/threonine-protein kinase 6-like | 54.20 | 75.20 | -0.17 | 0.7965 | not significant | PRKAA, AMPK |
| TRINITY_DN8133_c0_g3 | probable LRR receptor-like serine/threonine-protein kinase At1g63430 | 1.52 | 2.39 | -0.36 | 0.7998 | not significant | ------ |
| TRINITY_DN1867_c0_g1 | probable L-type lectin-domain containing receptor kinase S.5 | 27.14 | 40.40 | -0.26 | 0.8145 | not significant | IRAK4 |
| TRINITY_DN43686_c0_g1 | kinase family protein | 0.62 | 1.06 | -0.49 | 0.8328 | not significant | ------ |
| TRINITY_DN10265_c0_g1 | serine/threonine-protein kinase TOR-like | 5.09 | 7.29 | -0.28 | 0.8339 | not significant | MTOR, FRAP, TOR |
| TRINITY_DN2594_c0_g1 | probable LRR receptor-like serine/threonine-protein kinase At1g53430 | 26.53 | 42.90 | -0.20 | 0.8377 | not significant | ------ |
| TRINITY_DN6566_c0_g1 | probable leucine-rich repeat receptor-like serine/threonine-protein kinase At3g14840 | 9.47 | 14.24 | -0.28 | 0.8574 | not significant | ------ |
| TRINITY_DN8071_c1_g1 | chitinase 2-like | 0.79 | 1.41 | -0.60 | 0.8625 | not significant | ------ |
| TRINITY_DN6176_c0_g1 | putative concanavalin A-like lectin/glucanase domain-containing protein | 6.84 | 11.63 | -0.64 | 0.8681 | not significant | ------ |
| TRINITY_DN1303_c0_g2 | serine/threonine-protein kinase ATG1c-like isoform X1 | 8.82 | 12.11 | -0.20 | 0.8728 | not significant | ULK2, ATG1 |
| TRINITY_DN18063_c0_g1 | G-type lectin S-receptor-like serine/threonine-protein kinase At4g27290 isoform X2 | 3.88 | 5.65 | -0.10 | 0.9336 | not significant | ------ |
| TRINITY_DN8136_c0_g1 | putative tetratricopeptide-like helical domain-containing protein | 9.09 | 12.18 | -0.09 | 0.9372 | not significant | BSK |
| TRINITY_DN10575_c0_g2 | serine/threonine-protein kinase D6PKL2-like | 7.69 | 9.93 | -0.05 | 0.9544 | not significant | ------ |
| TRINITY_DN12117_c0_g2 | putative protein kinase-like domain, Concanavalin A-like lectin/glucanase domain protein | 2.92 | 3.79 | -0.05 | 0.9598 | not significant | IRAK4 |
| TRINITY_DN127103_c0_g1 | concanavalin A-like lectin/glucanase domain-containing protein | 1.34 | 1.70 | -0.10 | 0.9714 | not significant | ------ |
| TRINITY_DN80094_c0_g2 | probable serine/threonine-protein kinase At1g54610 | 8.35 | 10.52 | -0.06 | 0.9733 | not significant | CDK12_13 |
| TRINITY_DN26397_c0_g1 | probable LRR receptor-like serine/threonine-protein kinase At1g56130 | 25.18 | 31.33 | -0.11 | 0.9769 | not significant | ------ |
| TRINITY_DN21723_c1_g2 | glutathione transferase GST 23-like | 24.23 | 29.13 | -0.07 | 0.9835 | not significant | GST, gst |
| TRINITY_DN14011_c0_g2 | CBL-interacting serine/threonine-protein kinase 14-like | 7.84 | 10.21 | -0.02 | 0.9840 | not significant | SNF1 |
| TRINITY_DN12891_c0_g2 | hypothetical protein LSAT_7X58821 | 5.03 | 6.07 | -0.07 | 0.9860 | not significant | TNNI3K |
| TRINITY_DN1974_c0_g2 | serine/threonine-protein kinase CDG1 | 23.63 | 28.09 | -0.03 | 0.9940 | not significant | IRAK4 |
| TRINITY_DN24545_c0_g1 | serine/threonine-protein kinase-like protein CCR2 | 4.00 | 4.82 | -0.02 | 0.9971 | not significant | IRAK4 |
| TRINITY_DN38006_c0_g1 | probable LRR receptor-like serine/threonine-protein kinase At1g34110 | 1.75 | 2.47 | -0.01 | 0.9976 | not significant | ------ |
| TRINITY_DN92580_c0_g1 | endochitinase B1-like | 0.00 | 0.00 | 0.00 | 1.0000 | not significant | E3.2.1.14 |
| TRINITY_DN40930_c0_g2 | 40S ribosomal protein S10-1 | 0.00 | 0.00 | 0.00 | 1.0000 | not significant | RP-S10e, RPS10 |
| TRINITY_DN34202_c0_g1 | calcium-dependent protein kinase 12-like | 0.00 | 0.00 | 0.00 | 1.0000 | not significant | CAMK1;CAMK4 |
| TRINITY_DN29672_c0_g2 |  | 0.00 | 0.00 | 0.00 | 1.0000 | not significant | ------ |
| TRINITY_DN127290_c0_g1 | elongation factor 1-gamma-like | 0.00 | 0.00 | 0.00 | 1.0000 | not significant | EEF1G |
| TRINITY_DN33749_c0_g2 | protein EXORDIUM-like 3 | 0.00 | 0.00 | 0.00 | 1.0000 | not significant | ------ |
| TRINITY_DN4776_c0_g1 | serine/threonine-protein kinase STY46 | 41.63 | 28.74 | 0.93 | 0.0026 | not significant | ------ |
| TRINITY_DN12643_c0_g4 | G-type lectin S-receptor-like serine/threonine-protein kinase At4g27290 isoform X1 | 0.59 | 0.43 | 1.19 | 0.0215 | not significant | ------ |
| TRINITY_DN11229_c0_g1 | probable glutathione S-transferase parC | 15.32 | 9.69 | 0.83 | 0.0304 | not significant | GST, gst |
| TRINITY_DN14069_c0_g2 | L-type lectin-domain containing receptor kinase IX.1-like | 0.72 | 0.41 | 1.18 | 0.0383 | not significant | IRAK4 |
| TRINITY_DN16490_c0_g2 | kinesin-like protein KIN-14R | 3.48 | 2.69 | 0.72 | 0.0392 | not significant | KIFC2_3 |
| TRINITY_DN11405_c0_g1 | serine/threonine-protein kinase ppk15-like | 5.62 | 5.10 | 0.59 | 0.1751 | not significant | ------ |
| TRINITY_DN13713_c0_g1 | chitotriosidase-1-like | 2.38 | 1.22 | 1.14 | 0.4477 | not significant | E3.2.1.14 |
| TRINITY_DN7508_c0_g1 | LRR receptor-like serine/threonine-protein kinase FEI 2 | 4.16 | 2.77 | 0.90 | 0.5841 | not significant | ------ |
| TRINITY_DN10306_c0_g1 | probable serine/threonine-protein kinase At1g54610 | 2.46 | 2.66 | 0.26 | 0.6106 | not significant | CDK12_13 |
| TRINITY_DN114760_c0_g1 | putative endochitinase | 2.15 | 1.38 | 0.83 | 0.6257 | not significant | CHIB |
| TRINITY_DN1172_c0_g1 | chitotriosidase-1-like | 17.98 | 9.68 | 1.09 | 0.6694 | not significant | E3.2.1.14 |
| TRINITY_DN12418_c0_g1 | serine/threonine-protein kinase Aurora-1 | 9.13 | 8.58 | 0.44 | 0.6749 | not significant | AURKX |
| TRINITY_DN6441_c1_g1 | probable serine/threonine-protein kinase PIX7 isoform X1 | 28.56 | 33.25 | 0.26 | 0.7508 | not significant | IRAK4 |
| TRINITY_DN1071_c1_g1 | serine/threonine-protein kinase prpf4B | 24.29 | 23.47 | 0.31 | 0.7621 | not significant | ------ |
| TRINITY_DN8071_c0_g1 | chitinase 2-like | 49.93 | 31.22 | 0.88 | 0.7632 | not significant | ------ |
| TRINITY_DN6663_c0_g1 | serine/threonine-protein kinase D6PKL2-like | 12.54 | 8.98 | 0.71 | 0.7661 | not significant | ------ |
| TRINITY_DN60822_c0_g1 | probable receptor-like protein kinase At1g11050 | 25.20 | 17.05 | 0.76 | 0.8016 | not significant | ------ |
| TRINITY_DN9588_c1_g1 | serine/threonine-protein kinase STY46-like | 0.15 | 0.02 | 2.65 | 1.0000 | not significant | ------ |
| TRINITY_DN13971_c0_g3 | glutathione transferase GST 23-like | 17.90 | 13.67 | 0.59 | 0.8103 | not significant | GST, gst |
| TRINITY_DN16441_c0_g1 | endochitinase EP3-like | 0.39 | 0.39 | 0.55 | 0.8282 | not significant | E3.2.1.14 |
| TRINITY_DN44501_c0_g1 | L-type lectin-domain containing receptor kinase IX.1-like | 2.87 | 2.10 | 0.64 | 0.8441 | not significant | ------ |
| TRINITY_DN91000_c1_g1 | serine/threonine-protein kinase TOR-like | 9.73 | 9.81 | 0.25 | 0.8530 | not significant | MTOR, FRAP, TOR |
| TRINITY_DN12739_c0_g1 | probable inactive serine/threonine-protein kinase fnkC | 6.85 | 6.53 | 0.47 | 0.8621 | not significant | ------ |
| TRINITY_DN8414_c0_g1 | uncharacterized protein LOC110939657 | 3.26 | 3.80 | 0.18 | 0.8850 | not significant | ------ |
| TRINITY_DN13714_c0_g1 | putative 2S globulin, Glycoside hydrolase, catalytic domain-containing protein | 13.56 | 11.39 | 0.46 | 0.8854 | not significant | ------ |
| TRINITY_DN1071_c0_g2 | serine/threonine-protein kinase prpf4B | 19.73 | 23.64 | 0.08 | 0.8877 | not significant | PRPF4B |
| TRINITY_DN45356_c1_g1 | 40S ribosomal protein S10-A-like | 0.15 | 0.08 | 1.72 | 0.8921 | not significant | RP-S10e, RPS10 |
| TRINITY_DN13563_c0_g1 | glutathione transferase GST 23-like | 19.24 | 16.42 | 0.43 | 0.8994 | not significant | GST, gst |
| TRINITY_DN2439_c0_g4 | G-type lectin S-receptor-like serine/threonine-protein kinase At1g34300 | 16.79 | 15.30 | 0.36 | 0.8996 | not significant | IRAK4 |
| TRINITY_DN3945_c0_g1 | glutathione S-transferase U17-like | 71.95 | 66.75 | 0.30 | 0.9053 | not significant | GST, gst |
| TRINITY_DN13713_c0_g2 | acidic mammalian chitinase-like | 6.92 | 6.37 | 0.34 | 0.9085 | not significant | E3.2.1.14 |
| TRINITY_DN4127_c0_g2 | serine/threonine-protein kinase STE20-like | 20.65 | 23.76 | 0.11 | 0.9093 | not significant | ------ |
| TRINITY_DN29610_c0_g1 | putative protein kinase-like domain-containing protein | 1.41 | 1.32 | 0.34 | 0.9125 | not significant | ------ |
| TRINITY_DN18190_c0_g1 | probable serine/threonine-protein kinase PBL7 | 5.13 | 5.01 | 0.26 | 0.9158 | not significant | IRAK4 |
| TRINITY_DN3533_c0_g1 | leucine-rich repeat receptor-like serine/threonine-protein kinase At2g14510 | 7.21 | 6.55 | 0.35 | 0.9242 | not significant | IRAK1;IRAK4 |
| TRINITY_DN4881_c0_g2 | putative receptor protein kinase ZmPK1 | 28.18 | 29.45 | 0.22 | 0.9359 | not significant | IRAK4 |
| TRINITY_DN2415_c3_g1 | probable serine/threonine-protein kinase SIS8 | 6.92 | 8.11 | 0.10 | 0.9396 | not significant | ------ |
| TRINITY_DN4523_c0_g1 | glutathione S-transferase T1-like | 169.17 | 206.87 | 0.04 | 0.9415 | not significant | GST, gst |
| TRINITY_DN5343_c0_g1 | G-type lectin S-receptor-like serine/threonine-protein kinase At1g34300 | 63.61 | 73.80 | 0.12 | 0.9442 | not significant | ------ |
| TRINITY_DN14529_c0_g1 | beta-galactosidase 8-like isoform X1 | 4.17 | 4.44 | 0.17 | 0.9482 | not significant | ------ |
| TRINITY_DN9910_c0_g1 | hydroxyproline O-galactosyltransferase GALT2-like | 7.78 | 8.70 | 0.10 | 0.9492 | not significant | GALT2S |
| TRINITY_DN2879_c0_g2 | serine/threonine-protein kinase BLUS1-like isoform X2 | 12.60 | 14.71 | 0.07 | 0.9504 | not significant | OXSR1, STK39 |
| TRINITY_DN15615_c0_g1 | cysteine-rich receptor-like protein kinase 10 | 3.71 | 3.78 | 0.16 | 0.9552 | not significant | IRAK4 |
| TRINITY_DN6508_c0_g1 | protein SPIRRIG-like | 13.25 | 16.67 | 0.08 | 0.9564 | not significant | ------ |
| TRINITY_DN3024_c0_g1 | probable serine/threonine-protein kinase At1g54610 | 12.91 | 13.23 | 0.18 | 0.9575 | not significant | CDK12_13 |
| TRINITY_DN5021_c0_g1 | probable serine/threonine-protein kinase At1g54610 | 6.50 | 6.74 | 0.16 | 0.9602 | not significant | CDK12_13 |
| TRINITY_DN2304_c0_g1 | serine/threonine-protein kinase TOR-like | 12.40 | 13.99 | 0.08 | 0.9608 | not significant | MTOR, FRAP, TOR |
| TRINITY_DN10109_c0_g2 | L-type lectin-domain containing receptor kinase IX.1-like | 3.45 | 4.11 | 0.06 | 0.9665 | not significant | IRAK4 |
| TRINITY_DN21737_c0_g1 | probable glutathione S-transferase parC | 430.38 | 456.32 | 0.13 | 0.9716 | not significant | GST, gst |
| TRINITY_DN5437_c0_g1 | LRR receptor-like serine/threonine-protein kinase | 3.91 | 4.46 | 0.06 | 0.9744 | not significant | ------ |
| TRINITY_DN5775_c0_g3 | cold-responsive protein kinase 1-like | 6.26 | 7.37 | 0.11 | 0.9774 | not significant | IRAK4 |
| TRINITY_DN11195_c0_g1 | glutathione transferase GST 23-like | 60.26 | 64.65 | 0.09 | 0.9775 | not significant | GST, gst |
| TRINITY_DN43894_c0_g1 | putative sterigmatocystin biosynthesis protein stcT | 0.01 | 0.01 | 0.37 | 1.0000 | not significant | EEF1G |
| TRINITY_DN25824_c0_g1 | putative receptor-like serine/threonine-protein kinase | 11.87 | 12.58 | 0.09 | 0.9825 | not significant | IRAK1;IRAK4 |
| TRINITY_DN24229_c0_g1 | probable serine/threonine-protein kinase PBL11 | 5.65 | 6.19 | 0.08 | 0.9827 | not significant | IRAK4 |
| TRINITY_DN6776_c0_g1 | probable LRR receptor-like serine/threonine-protein kinase At1g63430 | 5.31 | 6.14 | 0.04 | 0.9839 | not significant | ------ |
| TRINITY_DN15265_c0_g1 | serine/threonine-protein kinase HT1-like | 20.24 | 24.71 | 0.03 | 0.9894 | not significant | ------ |
| TRINITY_DN34869_c0_g1 | serine/threonine/dual specificity protein kinase, catalytic domain-containing protein | 0.57 | 0.68 | 0.00 | 0.9988 | not significant | IRAK4 |
| TRINITY_DN24796_c0_g2 | serine/threonine-protein kinase HT1 | 0.01 | 2.86 | -7.70 | 0.0000 | down | SOS;LRRK2 |
| TRINITY_DN35093_c0_g1 | mannose/glucose-specific lectin-like | 10.09 | 179.57 | -3.77 | 0.0000 | down | ------ |
| TRINITY_DN54122_c0_g1 | PREDICTED: protein kinase and PP2C-like domain-containing protein | 0.02 | 1.21 | -5.43 | 0.0000 | down | ------ |
| TRINITY_DN64094_c0_g1 | Glutathione S-transferase/chloride channel, C-terminal | 1.32 | 12.70 | -3.29 | 0.0000 | down | GST, gst |
| TRINITY_DN77212_c0_g2 | 40S ribosomal protein S10-A-like | 0.16 | 3.77 | -3.98 | 0.0001 | down | RP-S10e, RPS10 |
| TRINITY_DN61708_c0_g1 | putative sterigmatocystin biosynthesis protein stcT | 0.10 | 1.43 | -3.83 | 0.0002 | down | EEF1G |
| TRINITY_DN100025_c0_g1 | elongation factor 1-beta-like | 0.15 | 1.79 | -3.26 | 0.0002 | down | EEF1B |
| TRINITY_DN30570_c0_g1 | chitinase | 0.32 | 4.45 | -3.45 | 0.0003 | down | ------ |
| TRINITY_DN32749_c0_g1 | hypothetical protein CARUB_v10016229mg | 0.11 | 3.14 | -4.67 | 0.0004 | down | ------ |
| TRINITY_DN84673_c0_g1 | hypothetical protein DM860_007840 | 0.07 | 1.56 | -4.12 | 0.0004 | down | ------ |
| TRINITY_DN49948_c0_g1 | serine/threonine-protein kinase SKM1-like | 0.00 | 1.13 | -5.78 | 0.0004 | down | CLA4 |
| TRINITY_DN73157_c0_g1 | serine/threonine-protein kinase hal4-like | 0.04 | 1.01 | -4.36 | 0.0047 | down | E2.7.11.- |
| TRINITY_DN3948_c3_g2 | nitrogen network kinase 1-like | 0.17 | 2.15 | -3.45 | 0.0049 | down | ------ |
| TRINITY_DN99642_c0_g2 | serine/threonine-protein kinase srk1-like | 0.03 | 1.53 | -5.19 | 0.0074 | down | ------ |
| TRINITY_DN28548_c0_g1 | PREDICTED: protein kinase and PP2C-like domain-containing protein isoform X1 | 0.10 | 1.84 | -4.02 | 0.0095 | down | ------ |
| TRINITY_DN45879_c0_g1 | CBL-interacting serine/threonine-protein kinase 7-like | 11.29 | 5.42 | 1.44 | 0.0002 | up | ------ |
| TRINITY_DN6521_c0_g3 | probable LRR receptor-like serine/threonine-protein kinase At1g67720 | 11.14 | 3.02 | 2.03 | 0.0023 | up | ------ |
| TRINITY_DN16164_c0_g2 | probable serine/threonine-protein kinase At4g35230 | 15.45 | 7.20 | 1.48 | 0.0038 | up | BSK |
| *Fold Change*, the ratio of expressions in *AMI* and *AMF* treatment of same gene; *q*-value, *p*-value adjusted by Benjamini and Hochberg method. | | | | | | | |

- 1. **Supplementary Figures**

**
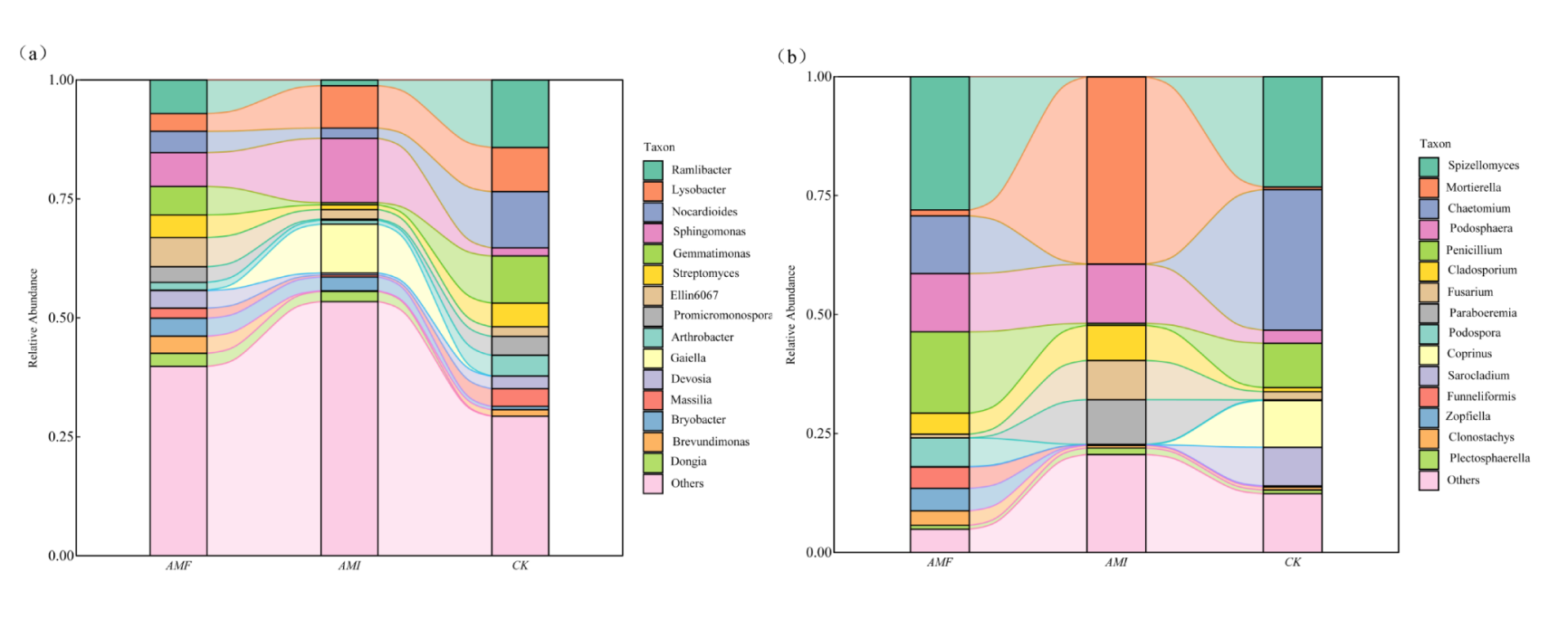
**

**Figure S1 Taxonomic profiles of soil microorganisms in three treatments at the genus level. (a) Bacteria; (b) Fungi.** Different colors represent different species. The proportion of colors on the stacking column represents the relative abundance of the corresponding species.


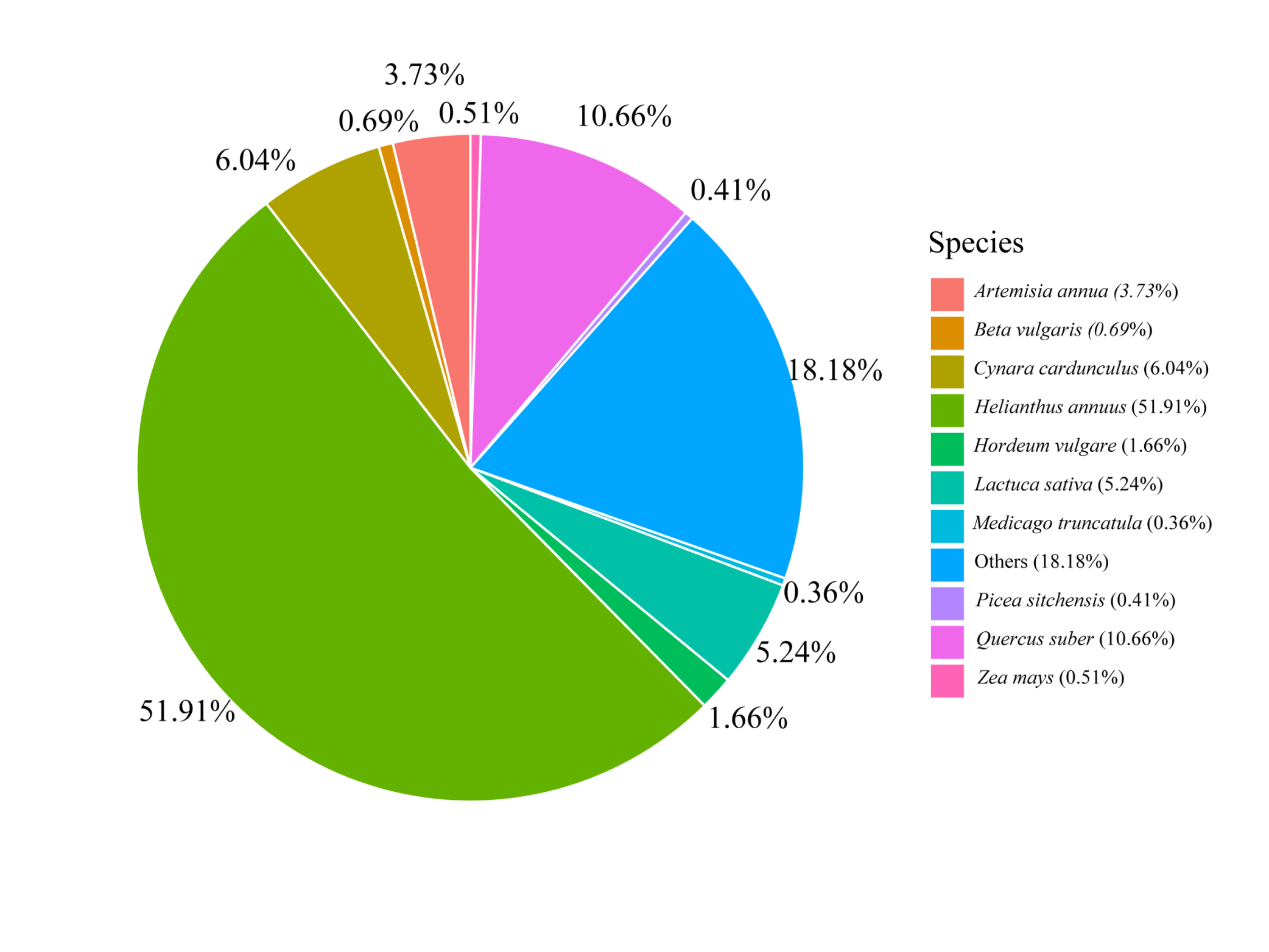
**Figure S2 Species distribution of matched proteins in NR database in *B. tripartita* transcriptome.** Each piece of fan indicates the number of matched proteins for various species.


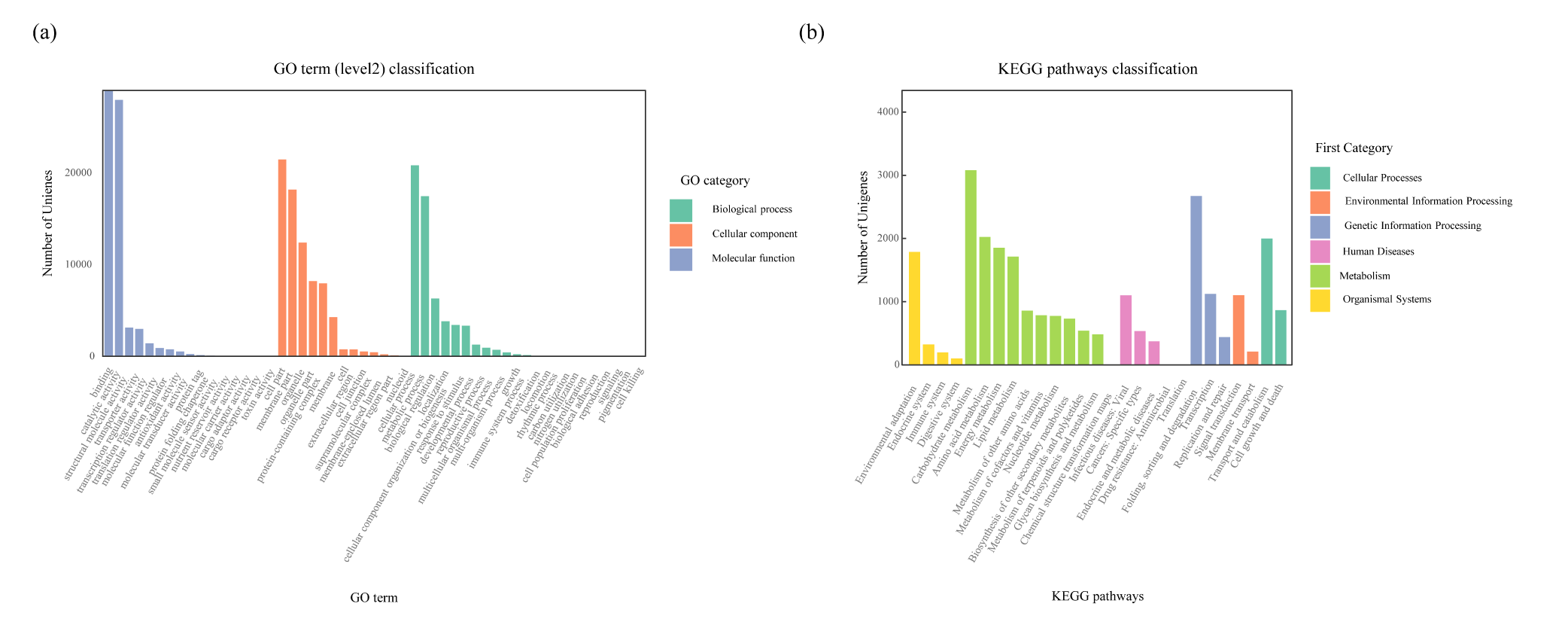
**Figure S3 (a) GO term (level 2) classification of the unigenes of *B. tripartita*. The x-axis indicates various GO terms;** The y-axis indicates the numbers of genes in GO terms; The color of column indicates the GO category to which GO term belongs； **(b) KEGG pathways classification of the unigenes of *B. tripartita*.** The x-axis indicates various pathways; The y-axis indicates the number of genes in a pathway; The color of column indicates the first category to which pathway belongs.
